# High-Throughput Phenotypic Screen to Identify FoxP3 Regulators in Primary T Cells

**DOI:** 10.1021/acschembio.5c01019

**Published:** 2026-04-03

**Authors:** Qian Wei, Ehsan Hajjar, Selma Cornillot-Clément, Elise Solli, Nuria García-Díaz, Johannes Landskron, Alexandra Gade, Rafi Ahmad, Kjetil Taskén

**Affiliations:** † Department of Cancer Immunology, Institute for Cancer Research, 155272 Oslo University Hospital, Oslo 0424, Norway; ‡ Norwegian Centre for Clinical Cancer Research, MATRIX, Division of Cancer Medicine, Oslo University Hospital, Oslo 0424, Norway; § Institute of Clinical Medicine, University of Oslo, Oslo 0318, Norway; ∥ Norwegian Centre for Molecular Biosciences and Medicine (NCMBM), Nordic EMBL Partnership, 6305 University of Oslo, Oslo 0318, Norway; ⊥ Department of Biotechnology, 3207 University of Inland Norway, Hamar 2317, Norway

## Abstract

FoxP3^+^ regulatory T cells (Tregs) maintain immune homeostasis by suppressing excessive antiself-immunity of effector T cells (Teffs) and thereby regulate autoimmunity and inhibit antitumor immune responses. Thus, Treg-targeting is a popular therapeutic strategy for both autoimmune diseases and cancer treatment. However, specific regulators of Tregs are needed to bypass adverse effects elicited by strategies that target molecules with overlapping expression between Tregs and other T cell subsets. We aim to identify small molecules that specifically target the key Treg lineage-defining transcription factor FoxP3. Here, we describe a high-throughput, flow-cytometry-based phenotypic screening assay, its data processing pipeline, and downstream methods for compound validation. We developed a set of algorithms to easily exclude compounds with high toxicity and to filter out autofluorescent compounds. Our screening assay is performed on human primary T cells by measuring FoxP3 levels in CD4^+^ T cells, using an automated liquid handling pipeline. Selected hit candidates from the screening were validated and assessed through functional analyses. From screening a library of approved drugs, we identified several candidates that regulate Treg functions through FoxP3. Seventeen chemical analogs of one hit compound were identified and characterized in cell-based assays, revealing structure–activity relationships and identifying compounds with more potent effects on the regulation of FoxP3 levels and Treg function. We conclude that this novel screening method could successfully identify FoxP3 regulators with an effect on Treg functions.

## Graphical abstract

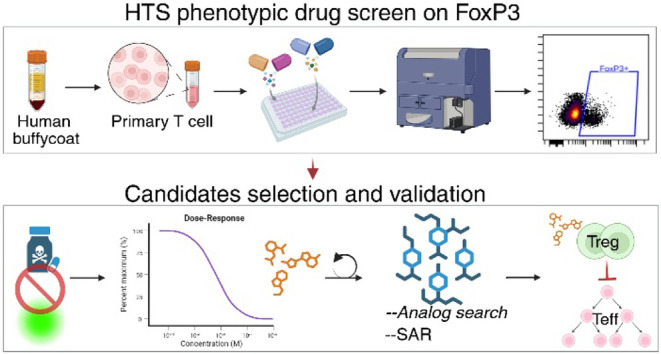

## Introduction

Regulatory T cells (Tregs) control immune homeostasis by suppressing excessive antiself-immunity of effector T cells (Teffs), thereby inhibiting antitumor immune responses and regulating autoimmunity. Recruitment of a high proportion of Tregs to the tumor microenvironment (TME) has been discovered in many cancer types, correlating with poor prognosis.^1,2^ In contrast, a decrease in the Treg population and loss of Treg functions lead to the development of autoimmune diseases such as rheumatoid arthritis,^3^ type I diabetes,^4^ myasthenia gravis,^5^ and multiple sclerosis (MS).^6^ Thus, targeting Tregs is a therapeutic strategy that is being explored for both autoimmune diseases^7^ and cancer treatment.^8^

It has been reported that depletion of Tregs could be an efficient approach to antitumor treatment, as seen in melanoma, pancreatic, and colorectal cancer models,^9^ suggesting that Treg depletion could be a potential therapeutic strategy. However, full depletion of Tregs in FoxP3^DTR^ mice can introduce severe autoimmunity^10^ or stimulate the development of pancreatic tumors due to alteration of associated fibroblasts,^11^ implying the need for drugs that modulate Tregs in a dose-dependent fashion without complete depletion for cancer treatment. In the context of autoimmune diseases, low doses of IL-2, which preferentially bind to the IL-2Rα (CD25) receptor on Tregs and facilitate Treg cell expansion, are the key Treg-targeting strategy to treat patients with autoimmune diseases, such as type I diabetes and systemic lupus erythematosus.^12–14^ In addition, infusion of Tregs has been tested in transplantation rejection (graft-versus-host disease), Crohn’s disease (TRIBUTE trial NCT03185000), or used to make CAR-Tregs for type I diabetes.^15^ However, there are technical challenges in Treg expansion, manufacturing, and potential cytotoxicity, as well as high cost.^16^

Currently, preclinical and clinical efforts targeting Tregs for cancer treatment are focusing on developing antibodies against surface markers that are highly expressed on Tregs, such as anti-CTLA-4, anti-PD-1, and anti-CD25 depleting antibodies, or targeting the signaling pathways in Tregs that regulate the expression of genes critical to Treg functions, such as PI3K^17^ and mTOR.^18^ However, this may introduce side effects due to overlapping expression of markers and shared signaling pathways among other immune cell types, including Teffs.^19,20^ There are some drugs that show increased frequency of Tregs and enhanced Treg suppressive functions suitable for autoimmune disease treatment, such as Glatiramer acetate in relapsing-remitting MS patients^21^ and IFN-β treatment in MS patients;^22^ however, the underlying mechanism of action is not clearly understood.

As the Treg lineage-specific transcription factor, Forkhead box protein P3 (FoxP3) plays a vital role in regulating genes necessary for Treg functions; it poses as a promising therapeutic target for drug discovery with potentially low side effects. Transcription factors have been considered nondruggable due to their small protein size and lack of specific targetable motifs or binding sites. However, FoxP3 has a benefit over other proteins due to its exclusive expression in Tregs rather than other cell types and organs, which can make it druggable.^23^ Nevertheless, the strategy to target FoxP3 still needs to be well-optimized due to the highly conserved Forkhead domain sequence shared among Forkhead families, especially FoxP proteins.^24^

With good membrane permeability and the ability to penetrate into the TME or tissues, small molecular compounds may be an optimal therapeutic option that provides good oral bioavailability and has low production costs compared to biologicals.^25^ Some efforts have been made to use pharmacologically available inhibitors targeting pathways that regulate both epigenetic and post- translational modifications of FoxP3, such as acetylation and methylation. Inhibition of histone/protein acetyltransferase p300 can impair Treg suppressive functions,^26^ while a more selective HDAC6 inhibitor is reported to promote Treg suppressive function in an autoimmune disease model.^27^ Inhibitors of protein arginine methyltransferase PRMT5 impaired the methylation of the FoxP3 protein, resulting in lower FoxP3 expression and enhanced antitumor response.^28^ However, these small molecules could also target other proteins regulated by the same pathways, implying toxicity or adverse effects.^25^ There are very few studies on the identification of direct regulators of FoxP3, with a peptide inhibitor^29^ and an antisense oligo that directly targets FoxP3 gene expression^30,31^ being the only published reports. Hence, there is still a strong need to identify small molecules to specifically target Tregs.

We have established an automated pipeline to perform high-throughput flow cytometry-based phenotypic screening for FoxP3 regulators in primary T cells, from which promising candidates that down-regulate FoxP3 and Treg functions were identified and further validated.^32,33^ Here, we provide a detailed description of the methods used for primary and secondary screening, the assay setup, data analysis, and hit validation. With the implementation of our well-defined data analysis tools, we could easily filter out false positives and toxic compounds. Characterization of promising hits and expansion with sublibraries of chemical analogs proved to be feasible for the identification of FoxP3 down-regulators from our screen.

## Results

### High-Throughput Flow Cytometry-Based Screen Assay Development and Quality Control

With the aim of identifying novel small molecules targeting Tregs, we established a screen using a flow cytometry-based assay tested on approved drugs in primary T cells. To perform the assay with high efficiency and accuracy, an automated pipeline was implemented for high-throughput flow-cytometry based screening (Figure 1). First, a Certus cell dispenser was used to get even distribution of cells into 384-well plates. After treatment with drugs, sample preparation and antibody staining were performed using a BioMek pipetting robot with an integrated centrifuge. Antibody dispensing was performed by an Echo550/650 acoustic dispenser to reduce variance among the wells. Finally, cells were run on a BD LSRFortessa with a high-throughput sampler (HTS) or a Sartorius iQue Screener PLUS flow cytometer, followed by data analysis in Cytobank or Forecyt (Figure S1A) to quantify FoxP3 expression in CD4^+^ T cells. Gating strategies are illustrated in Figure 2A and Figure S1B.

**Figure 1:**
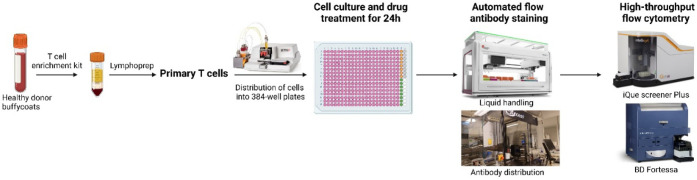
Pipeline for high-throughput flow cytometry-based screening of FoxP3 regulators. Primary T cells isolated from healthy donor buffy coats were distributed by the Certus Flex dispenser into 384-well plates, which were preprinted with compounds. The cells were treated with the compounds or DMSO at 10 μM for 24 h, with or without stimulation. Subsequently, the cells were stained with anti-CD4 and anti-FoxP3 antibodies using a Biomek i7 automated station and Echo550/650 acoustic dispenser. After the cells were washed, high-throughput flow cytometry was performed on either the iQue screener PLUS or BD LSRFortessa to measure FoxP3 expression in T cells. The workflow was generated in BioRender (https://BioRender.com).

**Figure 2:**
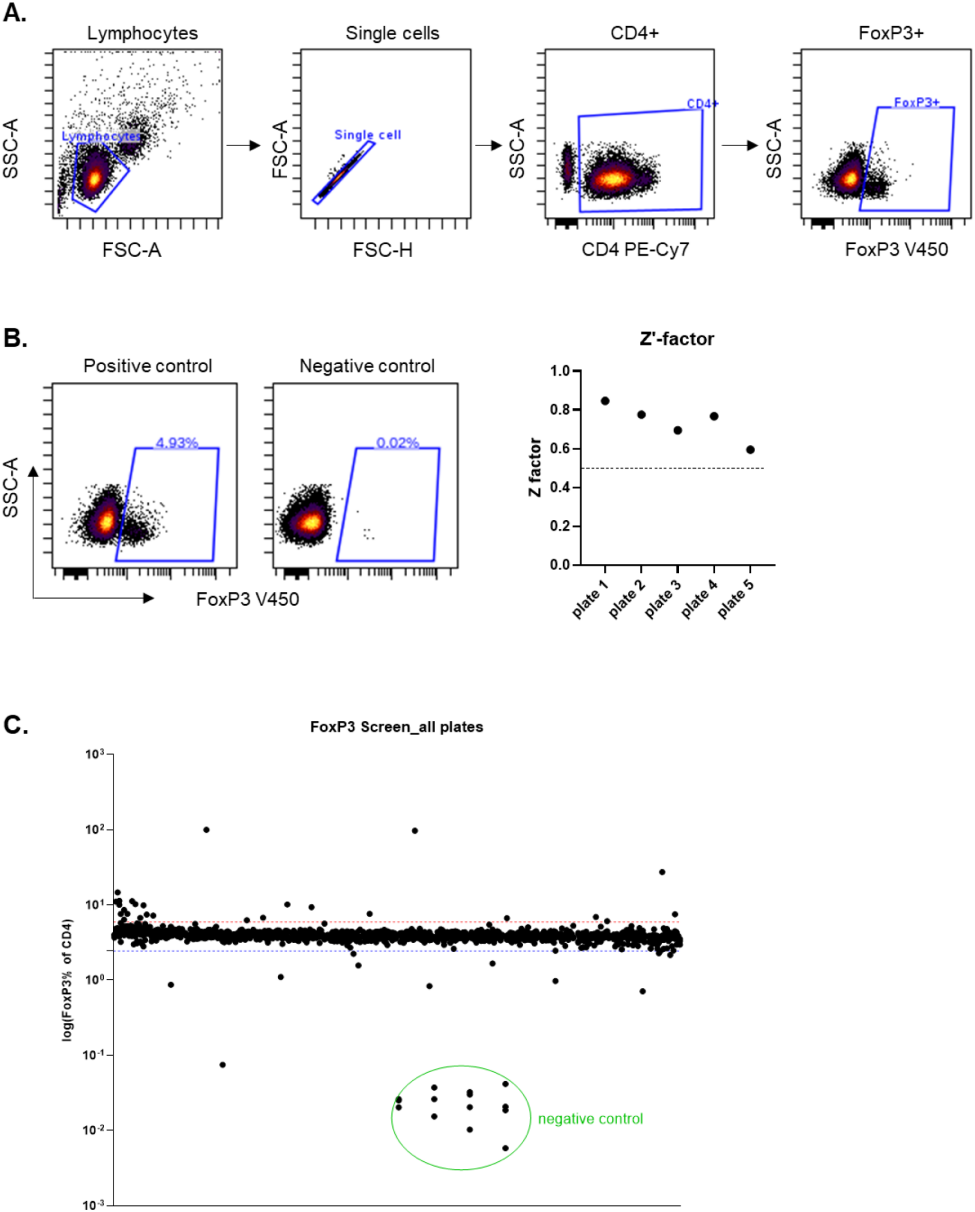
High-throughput flow cytometry-based phenotypic screen. CD3^+^ T cells isolated from healthy donors were distributed into 384-well plates preprinted with compounds for a 24 h drug treatment. Then, cells were fixed and permeabilized for anti-CD4 PE-Cy7 and anti-FoxP3 Horizon V450 antibody staining prior to high-throughput flow cytometry analysis. **A)** Gating strategy: FoxP3^+^ cells within CD4^+^ T cells were gated from singlets of lymphocytes. Data were analyzed in Cytobank. **B)** Left: Flow cytometry scatter plots show the representative gating of FoxP3 in CD4^+^ T cells from positive and negative controls used for Z’-factor calculation. Right: The Z’-factors of five plates in the screening of the Prestwick Chemical Library were calculated and plotted. **C)** Overview of the percentage of FoxP3 in CD4^+^ T cells from the Prestwick library screen is presented. Each dot on the graph represents an individual well from the screening plates. *Y*-axis: log10 scale of the FoxP3% in CD4^+^ T cells. The red and blue lines indicate ±50% deviation from the average value. Negative controls consisted of wells stained with the anti-CD4 antibody alone.

To screen the Prestwick Chemical Library (PCL, Greepharma) containing 1520 FDA/EMA-approved drugs, T cells were treated with compounds at a final concentration of 10 μM in a 384-well plate, with one column of wells containing DMSO-treated controls. The controls were stained with antibodies against either CD4 and FoxP3 or CD4 only, or left unstained (Figure S1C). For quality control, a Z’-factor^34^ was calculated for each plate based on the percentage of FoxP3^+^ cells in the CD4^+^ T cell population. Controls stained with the CD4 antibody only were used as negative controls, while controls with both CD4 and FoxP3 staining were used as positive controls (Figure 2B, Figure S2A and B). As shown in Figure 2B, the Z’-factors from the 5 plates in our pilot screen were all higher than 0.5, representing consistently high quality throughout the high-throughput screen. Therefore, we proceeded with data analysis and hit identification by selecting compounds that affected FoxP3 expression with at least a ±50% change compared to DMSO controls (Figure 2C, Figure S2C).

### Toxic Compound Exclusion

FoxP3 down-regulators may represent potential immunotherapies for cancer by inhibiting Treg functions. However, the drugs that showed inhibition of FoxP3 expression by flow cytometry could be the result of high toxicity in the cells. Therefore, as our screen was performed in primary T cells from healthy donors, the identification and exclusion of toxic compounds for further validation are important to avoid possible toxic effects on healthy immune cells. By analyzing the flow cytometry data, we observed that some wells with a lower percentage of FoxP3 than that of DMSO controls showed changes in the morphology of all lymphocytes (FSC vs SSC in flow cytometry plots), which were ultimately associated with cell death induced by toxic compounds (Figure 3A). Indeed, these morphology changes of lymphocytes for the representative compound wells can be captured as distinct FSC-H median intensity (MFI) compared to that of DMSO in the overlaid histograms (Figure 3B). FSC, forward scatter, is a measurement of light scattered in the forward direction in flow cytometry, whose intensity is proportional to the diameter of cells due to light diffraction. The change in FSC-A (forward scatter area) shown in Figure 3A and FSC-H (forward scatter height) in Figure 3B can both be supported as fundamental parameters to measure cell size. Specifically, FSC-A displays an integrated signal area under the pulse, which is directly proportional to cell size, while FSC-H shows the peak of signal intensity that is related to the narrowness of the cell pulse. Thus, in Compound 2- and Compound 3-treated lymphocytes, both FSC-A and FSC-H represent clear morphology changes due to the introduced toxicity (Figure 3A and B). Notably, compounds with deviant FSC-H MFI were verified to show different toxicity patterns at 10 μM by viability staining (Figure 3C), at the same concentration tested in Figure 3B. Therefore, we could easily identify the compounds with potential high toxicity by normalizing the FSC-H MFI of each compound well to that of DMSO (Δratio) in each screening plate (Figure 3D). For example, Compound 3 showed a much higher Δratio than average, while Compound 2, with moderate toxicity, shows a slightly higher Δratio than average, illustrated by their outlier in the Δratio plot of each individual plate. Interestingly, Compound 3 showed significant toxicity at 1 μM (Figure 3C), which confirmed a correlation between the Δratio value and toxicity. Compounds with no toxicity, such as Compound 1, showed a similar Δratio to the screening plate average.

**Figure 3:**
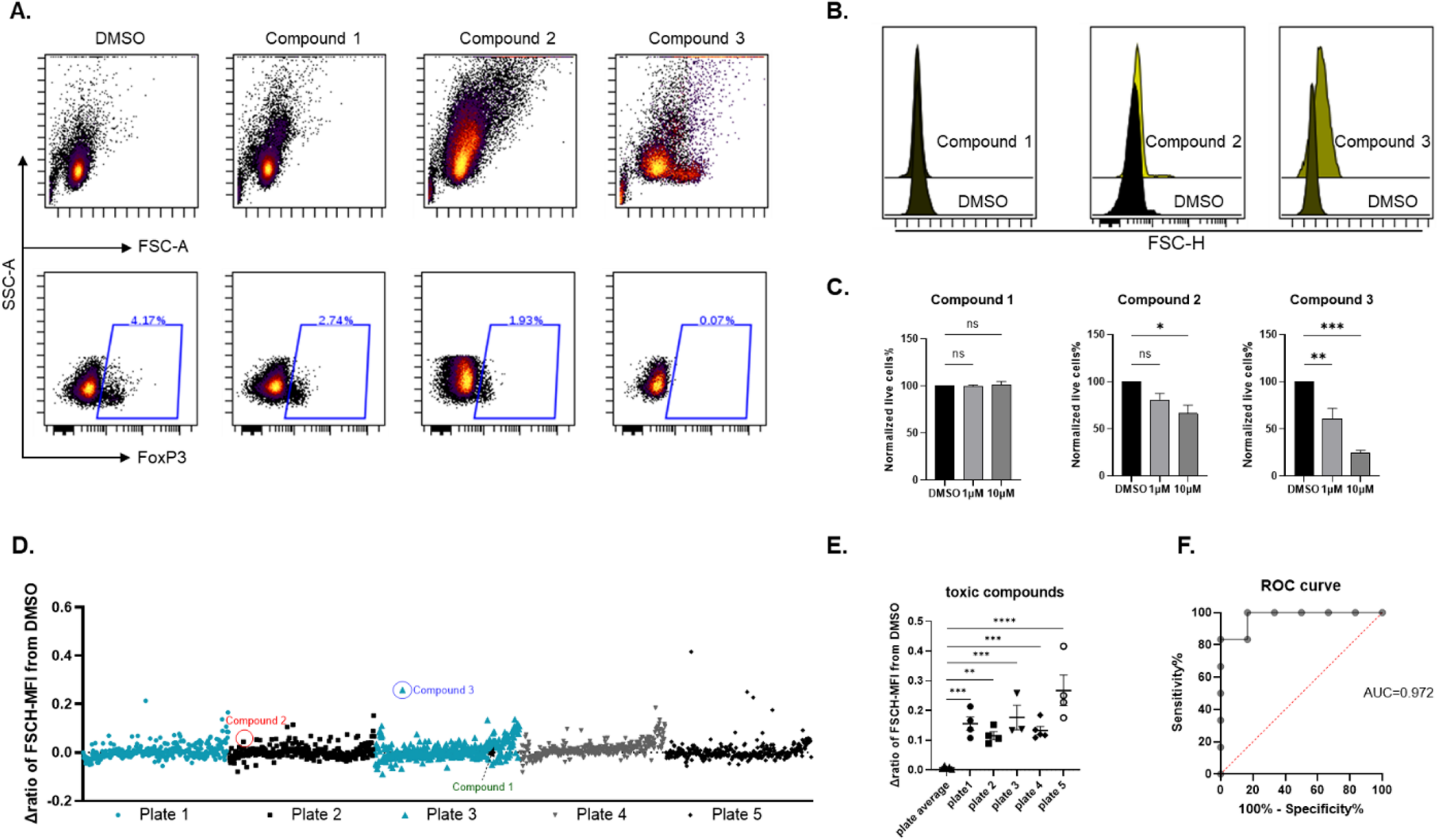
Exclusion of toxic compounds. (**A–B)** Flow cytometry data from the screen, as performed in Figure 2 were analyzed. (A) Representative flow cytometry plots for the DMSO control and compounds that reduce FoxP3% are shown. Upper: scatter plots for all lymphocytes in the indicated wells. Lower: FoxP3 gating showing how FoxP3 levels were observed in CD4^+^ T cells. (B) Histogram overlays of FSC-H for all lymphocytes comparing DMSO with each individual compound were presented. **C)** CD3^+^ T cells were treated with either DMSO or the specified compound at 1 and 10 μM for 24 h, followed by viability dye Ax700 staining to assess the percentage of live cells by flow cytometry analysis. The percentage of live cells in the compound-treated samples was normalized to DMSO control. The data are represented as mean ± SEM. * *p* < 0.5, ** *p* < 0.05, *** *p* < 0.005, ns: not significant. (*n* = 3 healthy donors, one-way ANOVA). **D)** The plot showing the median fluorescence intensity (MFI) of FSC-H, calculated as Δratio of each compound-treated well to DMSO controls, from five plates in the Prestwick Chemical Library screen is displayed. Representative compounds are highlighted in different colors. **E)** Toxic compounds were selected using a cutoff of ±3D of each screening plate average, using FSC-H MFI Δratio data from **(D).** The statistics were determined by comparing the ratio of selected compounds in each plate to that of the averages from the whole screening. ** *p* < 0.05, *** *p* < 0.005, **** *p* < 0.0005. (*n* = 3 healthy donors, 2-way ANOVA). **F)** ROC analysis was performed in GraphPad by comparing the FSC-H Δratio and viability stain data in the same sets of compounds.

Importantly, using Δratio data for each compound and a cutoff of three standard deviations (±3SD) from the means of each screening plate, we could easily find the outlined compounds that are toxic (Figure 3E). The ROC analysis, by comparing the FSC-H Δratio with viability staining, verified the good performance of the algorithm (Figure 3F). This analysis can therefore serve as a simple and efficient tool to identify compounds with potential toxicity in healthy cells by capturing the toxicity-induced morphology changes using FSC-H MFI. Further validations will thus be easy to proceed with by excluding toxic candidates.

### Filtering Out Autofluorescent Compounds

FoxP3 up-regulators identified from our screen could also be applied for autoimmune disease treatment. By analyzing the gating of potential FoxP3 up-regulators, we surprisingly found that high FoxP3% in some wells showed a distinct staining pattern in the FoxP3-V450 channel present as a FoxP3^+^ population change (Figure 4A). Compounds 4 −7 have higher FoxP3% compared to DMSO, and Compounds 6 and 7 clearly demonstrate this population shift in the gating (Figure 4A). Indeed, Compound 6 introduced a shift of the whole population in the V450 channel, representing nearly 100% of the FoxP3^+^ population, while the population shift in Compound 4 was not obvious. These whole population shifts are most likely due to the autofluorescent properties of the compounds that result in false-positive signals in the flow cytometry analysis. In fact, many fluorescent compounds have been reported to absorb light in the range from 355 to 488 nm and/or emit in the range of 350–550 nm,^35^ which resulted in the disturbance or loss of the V450 signal coming from the FoxP3 antibody in our flow cytometry staining. Therefore, to filter out the autofluorescent compounds and determine the actual FoxP3 up-regulators, we established a tool to distinguish false positives. As shown in Figure 4B, the V450 MFI stain ratio for each compound in the Prestwick library screen was obtained by dividing the V450 MFI of FoxP3^+^ populations by that of the FoxP3^–^ populations within the same plate. Interestingly, V450 MFI ratios of most wells are close to the average value of each plate, with some outliers having either higher or lower ratios. Compounds with similar V450 MFI ratios to the plate average, like Compound 4 in plate 5, which showed a high percentage of FoxP3^+^ cells, suggested a real up-regulating effect of the compound on FoxP3 expression (Figure 4A and B). In contrast, Compound 5, Compound 6, and Compound 7 showed distinct V450 MFI ratio patterns deviant from their plate averages. Indeed, Compound 5 and Compound 7 (marked in red or purple circles in Figure 4B) have lower V450 MFI ratios compared to the average, which represented moderate population shifts as shown in Figure 4A, while the V450 MFI ratio of Compound 6 (marked in a blue circle in Figure 4B) was an extreme outlier that is extensively higher than the average, showing nearly a complete whole population shift and high autofluorescence in Figure 4A. To determine the threshold for autofluorescent compound filtering, data from the validation of 15 compounds concluded that a cutoff using ±3SD of the plate average V450 MFI ratio is sufficient to identify compounds with high autofluorescence. In addition, compounds with low or moderate autofluorescence can be distinguished with at least a −1SD or −2SD change from the plate average (Figure 4C).

**Figure 4:**
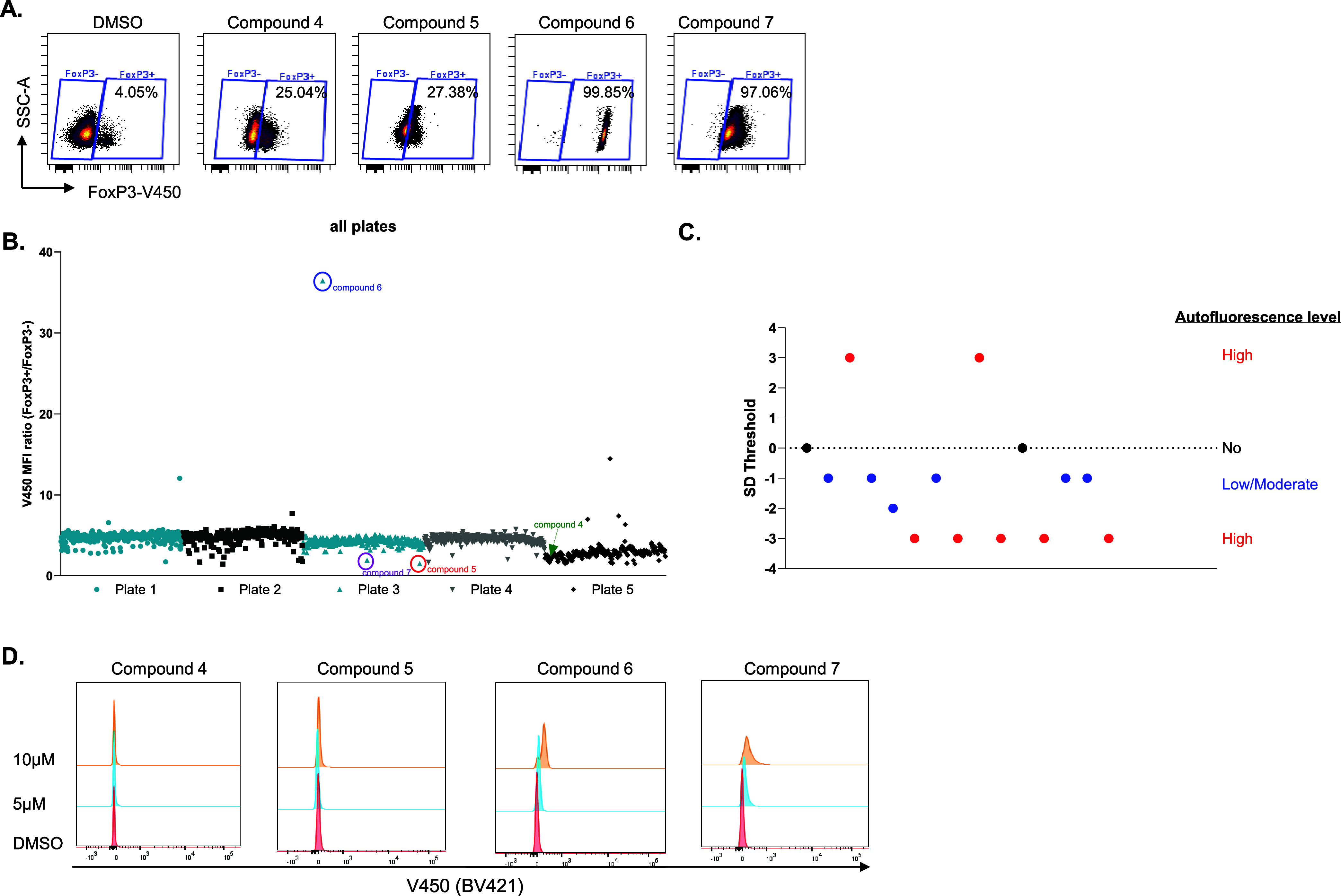
Filtering out compounds with autofluorescence. **A)** Representative pseudocolor plots show the gating of FoxP3^+^ and FoxP3^–^ cells within CD4^+^ T cells from compound-treated wells, which exhibited higher FoxP3 levels compared to the DMSO control. **B)** The plot of the V450-MFI ratio from five plates in the Prestwick Chemical Library screen is shown. Each dot represents an individual well in the screening plate, excluding wells without antibody staining. The ratio (*Y*-axis) was calculated by dividing the V450 MFI of FoxP3^+^ populations by that of the FoxP3^–^ populations. Representative compounds are indicated in different colors. **C)** The plot summarizes the threshold determination by analyzing 15 compounds. The threshold is divided into different groups: +3SD, −3SD, −2SD, −1SD, and 0SD of each plate average. Colored dots represent compounds with different autofluorescence levels: red, high; blue, low/moderate; black, no autofluorescence. **D)** CD3^+^ T cells were treated with either DMSO or the compounds at 5 or 10 μM for 24 h, followed by flow cytometry analysis with all channels enabled on the BD LSRFortessa. The histogram overlays represent changes in intensity in the V450 channel for cells treated with the compounds compared with the DMSO control.

Furthermore, we developed a method to examine the autofluorescent properties of the selected compounds by flow cytometry analysis. T cells were treated with compounds only, without antibody staining, before analysis in a BD LSRFortessa flow cytometer with all channels open (Figures S3–S6). Consistently, in channel V450 (BV421), which was used for the screen, treatment with Compound 5, Compound 6, and Compound 7 at 10 μM introduced distinct population shifts compared to DMSO (Figure 4D, Figures S4A, S5A, and S6A), while no population shift was observed in Compound 4-treated cells (Figure 4D and Figure S3A). Importantly, the selected compounds were further validated by changing the FoxP3 antibody fluorochrome from V450 to Ax647 to avoid the disturbance of signals in the flow cytometry analysis caused by compound autofluorescence, which resulted in a different staining pattern of FoxP3 in cells treated with autofluorescent compounds (Figures S4B, S5B and S6B). For the non-autofluorescent Compound 4, the staining pattern of FoxP3 persisted regardless of the fluorochrome (Figure S3B).

The spectral shift in the V450 (BV421) channel introduced by autofluorescent compounds is consistent with their potential properties to absorb light in the range of 355 to 488 nm and/or emit in the range of 350–550 nm.^35^ These results verified that the V450 MFI ratio change is consistent with the spectral shift in the V450 (BV421) channel when identifying autofluorescent compounds, suggesting a reliable tool using the cutoff threshold stated in Figure 4C. Therefore, we concluded that compounds with autofluorescent properties can be identified by the deviation of the V450 MFI ratio from the plate average and easily filtered out as false-positive hits. These compounds can, however, be further validated using optimal fluorochrome-conjugated antibody panels.

### Hit Validation in Cell-Based Assays

To keep the high-throughput screen cost-effective and efficient, the primary screen was performed on pooled T cells from different donors without replicates. Potential hit candidates selected from the above analysis, therefore, needed to be validated in different individual healthy donors to determine their actual effects on FoxP3 regulation.

First, Compound 4 was confirmed to introduce a higher FoxP3% in CD4^+^ T cells at 30 μM compared to the DMSO control (about 50% higher) in both naïve (unstimulated) and TCR-stimulated T cells (Figure 5A and Figure S7). Importantly, Compound 4 (vitamin D3) was reported to be an up-regulator of FoxP3,^36^ which served as a promising validation of our screen for selecting FoxP3 regulators.

**Figure 5:**
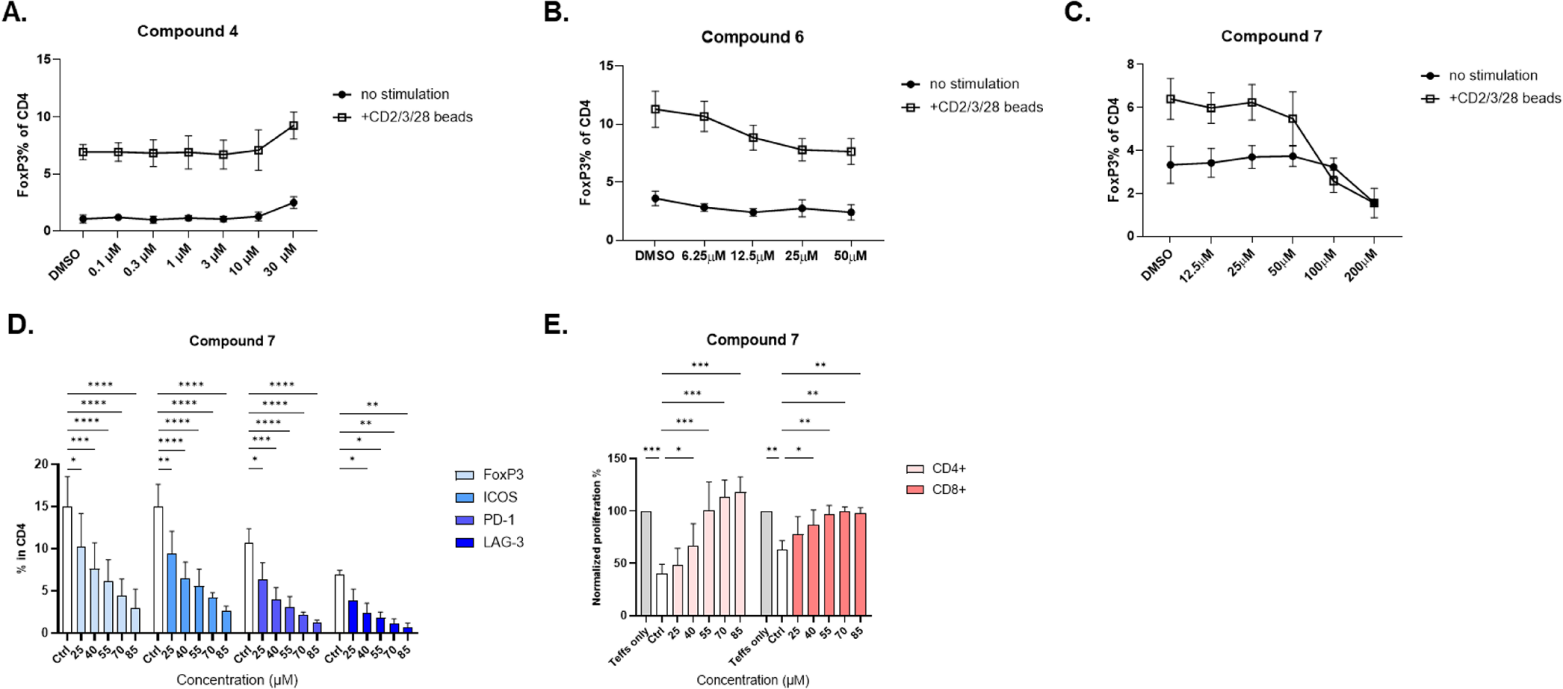
Compounds validation in cell-based assay. (**A–C)** CD3^+^ T cells were treated with the specified compounds with or without TCR stimulation (anti-CD2/CD3/CD28 beads) at the indicated concentrations for 24 h. Cells were then stained with viability dye Ax700, followed by flow cytometry analysis of the percentage of FoxP3 in live CD4^+^ T cells. **D)** CD3^+^ T cells were treated with Compound 7 at the indicated concentrations for 48 h under TCR stimulation. The expression of Treg markers was assessed through flow cytometry analysis by gating on CD4^+^ FoxP3^+^ T cells. For TCR stimulation, anti-CD2/CD3/CD28 beads were added to the cells in a 1:2 ratio. **E**) Tregs were treated with Compound 7 for 48 h and then cocultured with CellTrace Far Red-stained Teffs for 4 days. The proliferation of CD4^+^ and CD8^+^ Teffs was determined by measuring the percentage of CellTrace Far Red^+^ cells among live cells, which was normalized to the Teff-only controls. Data are represented as mean ± SEM (*n* = 3 healthy donors). * *p* < 0.5, ** *p* < 0.05, *** *p* < 0.005, **** *p* < 0.0005, 2-way ANOVA.

Second, to examine the effects of autofluorescent compounds shown as examples in Figure 4, we optimized the antibody panel for each compound to avoid any disturbance caused by autofluorescence in the flow cytometry analysis. This optimization was achieved by running compound-treated cells in flow cytometry with all channels open. For example, Compound 5 exhibited signal increases in BV421, BV510, and BV605 channels compared to DMSO (Figure S4A). Thus, these channels could not be used for flow cytometry staining. By using the FoxP3-Ax647 antibody instead, however, Compound 5 resulted in no effect on FoxP3 regulation (Figure S4B), which supported our above filter tool for identifying compound autofluorescence.

Interestingly, when the optimal flow cytometry staining panel was determined for Compound 6 and Compound 7 (Figures S5 and S6), we observed down-regulation effects of these compounds on FoxP3 in both naïve and TCR-stimulated T cells (Figure 5B and C, Figure S7).

With the identification of Compound 7 (ethoxyquin) as an effective FoxP3 down-regulator, we further examined its effects on T cells at a lower concentration range during TCR stimulation. Interestingly, Compound 7 exhibited a significant reduction in the expression of all Treg markers we tested, in addition to FoxP3 (Figure 5D). Functional studies using our well-established Treg suppression assays revealed that treatment with Compound 7 in Tregs inhibited their suppressive functions toward Teffs, which significantly restored both CD4 and CD8 Teff proliferation (Figure 5E).

### Hit Expansion by Searching and Validation of Compound Analogs

Compound 7, identified as the synthetic antioxidant ethoxyquin, was thus confirmed to down-regulate FoxP3 and Treg function in our cell-based assay. To further evaluate the efficiency and mechanism of ethoxyquin, compound analogs were identified, and three of these were initially tested in cell-based assays (Figure 6A). Interestingly, all three analogs showed greater effectiveness in down-regulating FoxP3 expression compared to the original compound ethoxyquin, with EA2 and EA3 having IC50 values lower than 10 μM (Figure 6B). Importantly, none of these compounds showed cytotoxic effects in T cells at the tested concentrations (Figure 6C).

**Figure 6:**
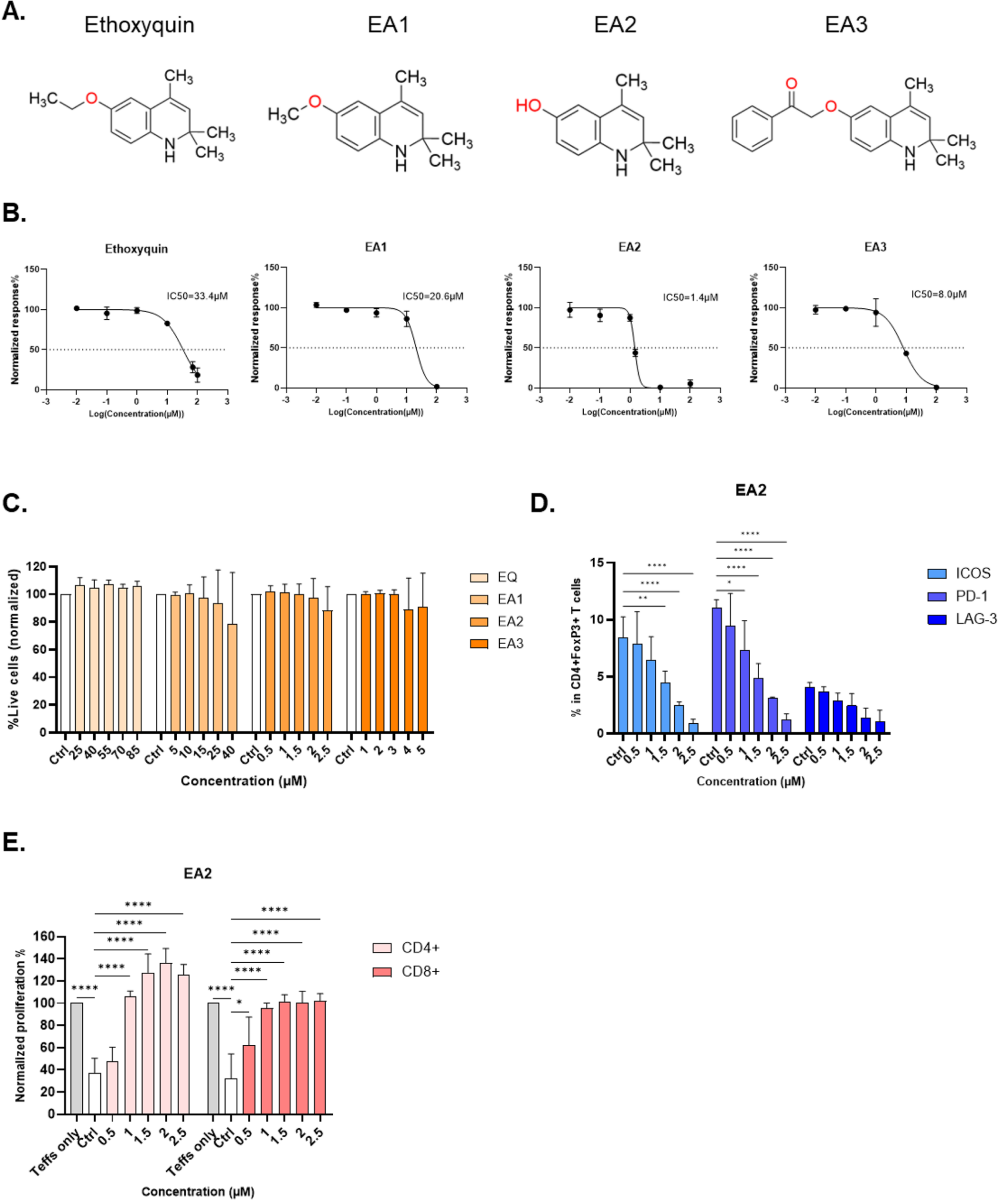
Validation of ethoxyquin analogs in cell-based assay. **A)** The compound structures of the original hit ethoxyquin and its three analogs, EA1–EA3 are presented. **B–C)** CD3^+^ T cells were treated with compounds under TCR stimulation at the indicated concentrations for 48 h, followed by flow cytometry analysis. (B) Normalized response: the percentage of FoxP3 in CD4^+^ T cells in the compound-treated samples was normalized to the DMSO control, which was set to 100%. IC50 values were determined by best-fit dose–response curves in GraphPad prism. (C) The percentage of live cells among single cells in the compound-treated samples was normalized to the DMSO control, which was set to 100%. **D)** CD3^+^ T cells were treated with compound EA2 under TCR stimulation at different concentrations for 48 h, followed by flow cytometry analysis to examine the expression of ICOS, PD-1, and LAG-3 in CD4^+^ FoxP3^+^ T cells. **E)** Tregs treated with compound EA2 for 48 h were subsequently cocultured with CellTrace Far Red-stained Teffs for 4 days under TCR stimulation. The proliferation of CD4^+^ and CD8^+^ Teffs was measured by the percentage of CellTrace Far Red^+^ cells among live cells, which was normalized to that of Teff only. Data are represented as mean ± SEM (*n* = 3 healthy donors). * *p* < 0.5, ** *p* < 0.05, **** *p* < 0.0005, 2-way ANOVA.

Using the more potent compounds derived from the original hit, another round of analog searches was conducted based on the structures of EA2 and EA3. This yielded eight compounds (Figure S8A) and six compounds (Figure S8B) respectively. When tested in T cells, EA7, EA9, and EA11, derived from EA2, showed similar down-regulating effects on FoxP3, while all analogs derived from EA3 (EA15–EA20) showed inhibitory effects at most concentrations (Figure S9A). However, many compound analogs also exhibited high toxicity in T cells at higher concentrations (Figure S9B). We concluded that, in addition to EA2 and EA3, EA7 -having an IC50 on FoxP3 at 2.6 μM- and EA15- with an IC50 of 1.9 μM -were the most promising ethoxyquin analogs that did not introduce toxicity in T cells (Figure S9 and Table 1).

**Table 1:**
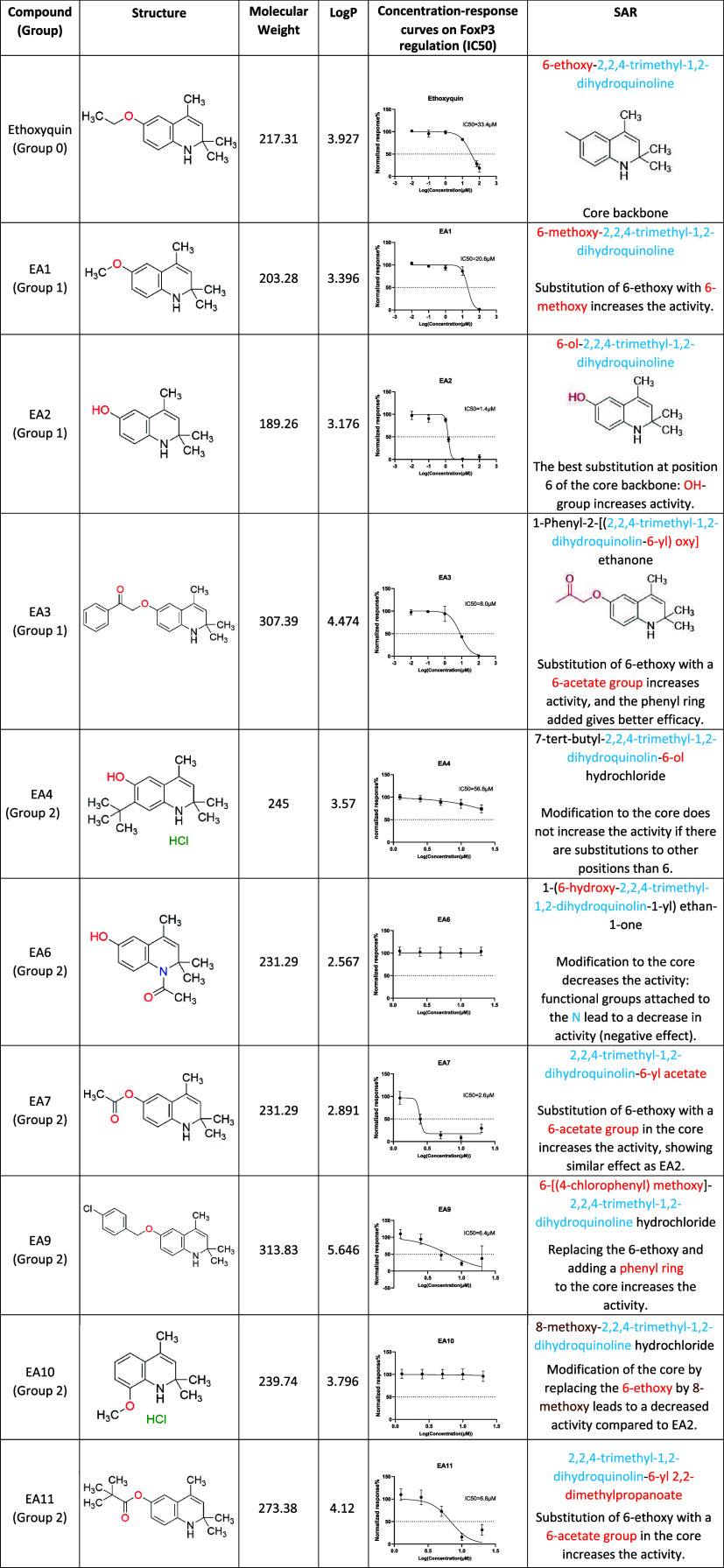

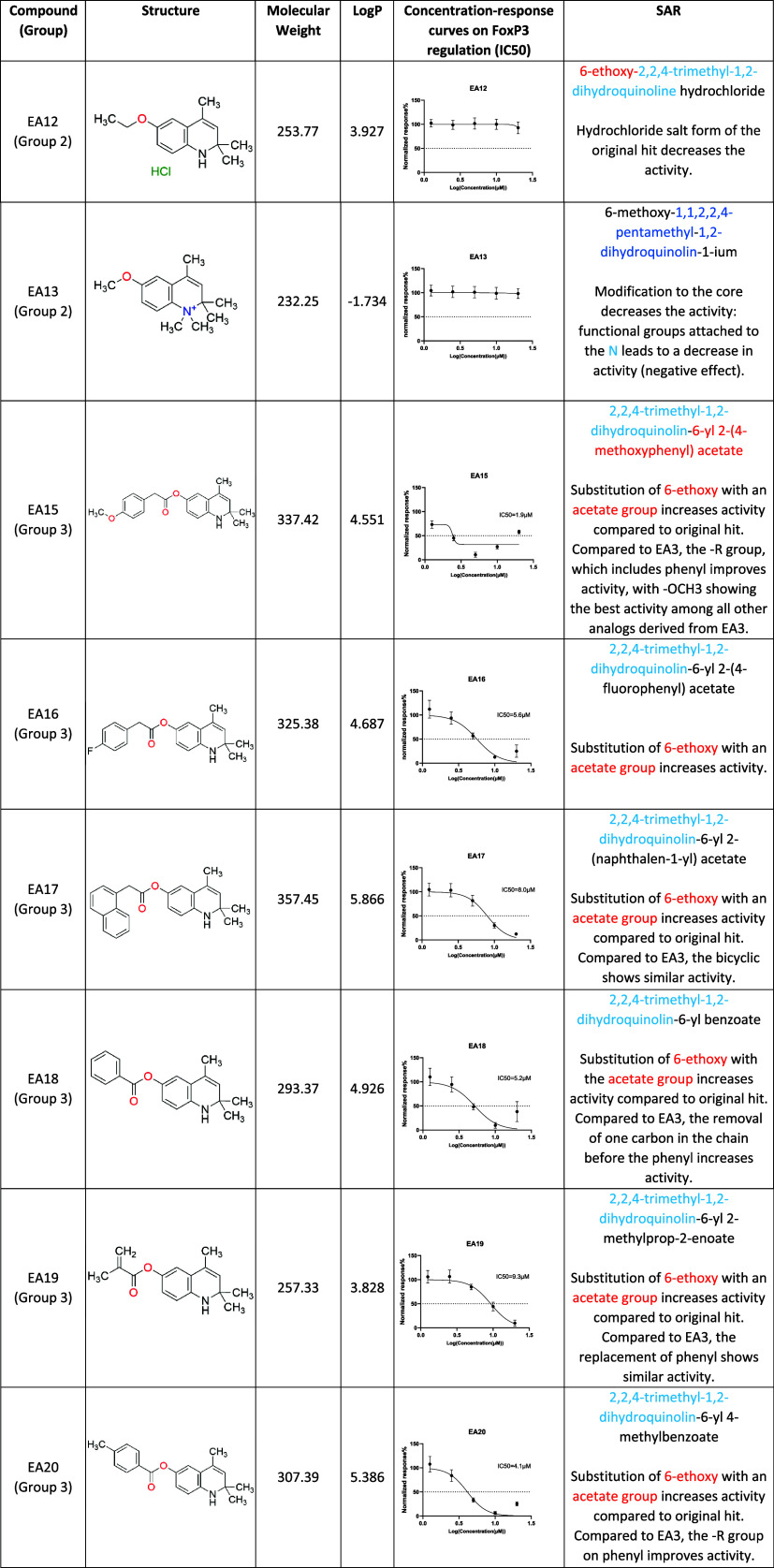
Summary of SAR Information for Ethoxyquin and Its Analogs^a^

a To obtain concentration–response curves, CD3^+^ T cells were treated with compounds under TCR stimulation at the indicated concentrations for 48 h, followed by flow cytometry analysis. The concentration–response curves were calculated based on normalized response, which was determined using the percentage of FoxP3 in CD4^+^ T cells in the compound-treated samples, normalized to the DMSO control that was set to 100%. IC50 was determined with best-fit values by calculating log­(inhibitor) vs normalized response in GraphPad Prism (*n* = 3 healthy donors).

To further validate the functions of the most promising analog, EA2 was also confirmed to inhibit the expression of Treg markers (Figure 6D) and restore Teff proliferation (Figure 6E) at its IC50 concentration. Ethoxyquin and EA2 inhibition of Treg suppression of antigen-specific Teff was also observed (Figure S10A). Notably, neither ethoxyquin nor EA2 affected other non-T cell populations, suggesting a selective effect on FoxP3 in Tregs (Figure S10B). Interestingly, ethoxyquin and EA2 exhibited significant suppression at both the protein and mRNA levels of FoxP3 (Figure S10C,D and E). This provides a foundation for understanding the structure–activity relationships of ethoxyquin-like compounds in FoxP3 regulation.

### Structure–Activity Relationship (SAR) of Ethoxyquin-Like Compounds

Examination of the efficacy of all ethoxyquin analogs in cell-based assays provides a foundation for understanding the SAR of ethoxyquin-like compounds that modulate FoxP3 expression. In Table 1, we show a summary of the SAR information for ethoxyquin and tested analogs, categorized into different groups: Group 0 for the original hit, Group 1 for the first-round analog search based on ethoxyquin, Group 2 for analogs derived from EA2, and Group 3 for analogs derived from EA3. The core backbone text is highlighted in blue, with changes marked in red for the start of the *in-silico* searches. Indeed, substitution of the core backbone 2,2,4-trimethyl-1,2-dihydroquinoline at position 6 with an −OH group, which represents EA2, showed the highest activity among all compounds. The analogs derived from EA2 showed similar effects as EA2 when the 6-ethoxy group was substituted by a 6-acetate group (EA7 and EA11) or when a phenyl ring was added (EA9). The substitution of 6-ethoxy in the core with a 6-acetate group also significantly increased the activity compared to ethoxyquin, as represented by EA3 and Group 3 analogs. Notably, modifications of the core backbone with functional groups attached to the N lead to a decreased effect in FoxP3 down-regulation or no effect (EA6 and EA13). In conclusion, the substitution of 6-ethoxy in the core is the key modification of ethoxyquin that affects analog activity on FoxP3 expression, resulting in down-regulation, among which the 6-OH group or 6-acetate group substitutions correlated most strongly with enhanced activity (Figure S11).

## Discussion

Transcription factors are generally considered undruggable; thus, a phenotypic screen that utilizes effective functions in the cell as a readout is the main method to identify regulators of transcription factors and provide potential hits with biological actions. Our phenotypic flow cytometry analysis could help identify potential compound candidates with biological functions inside the cells that regulate FoxP3 expression directly or indirectly.

The initial drug screen was performed in T cells treated with compounds at a single concentration (10 μM) for 24 h, which was optimized based on our previous observation that MEK inhibitors exhibited efficient inhibition of FoxP3 expression at 10 μM without inducing cell toxicity,^37,38^ and that a 24 h treatment of nonstimulated T cells with indomethacin was optimal to down-regulate Treg function.^39^ Additionally, using mixed T cells from different donors can help to average effects, as we observed minimal donor-dependent variance.^38^ These optimizations aim to conduct a high-throughput drug screen cost-effectively. Our pilot screen was designed to identify regulators controlling endogenous FoxP3 levels, which are normally lowly expressed in nonstimulated primary T cells. Therefore, we observed fewer down-regulators than up-regulators in the pilot screen due to the low starting values of FoxP3. A moderate stimulation of T cells with anti-CD2/CD3/CD28 beads to obtain higher basal levels of FoxP3 expression can provide a more robust signal and be suitable for candidate validation.^32^

Removal of false positive and false negative regulators is necessary to clean up the preliminary hit list for further validation. First, hit compounds should be tested for their toxicities in serial concentrations in primary cells from healthy donors, especially those showing a reduction in FoxP3 expression. The most important side effects to avoid when targeting Tregs should be the lack of Treg specificity (vs Teffs) and toxicity, aiming for disease control without disturbing immune cell homeostasis. As Tregs are a subset of T cells, the toxicity to Tregs can also affect Teffs due to the shared signaling pathways for cell growth. Therefore, it is necessary to discard the drugs demonstrating high toxicity in non-Treg T cells from healthy donors while keeping the ones with more specificity or toxicity in Tregs over other immune cell subsets. The implementation of our analysis tools helps to exclude toxic compounds that affect T cells in general without the need for a viability dye stain in the screening, making the process both efficient and cost-effective. In general, we could set the Δratio change thresholds as ±3SD changes from DMSO, which is sufficient to exclude most false positive hits on FoxP3 downregulation; however, this approach may result in compound hits with potential moderate to high toxic properties.

Notably, compounds with possible autofluorescent properties may be down-regulators if tested in a proper flow cytometry staining panel. For example, we found that Compound 6 was actually a FoxP3 down-regulator in our validation, even though it showed misleadingly high FoxP3 expression due to autofluorescent properties. Therefore, each compound selected from the phenotypic screen should be examined carefully by determining its own optimal staining panels to account for autofluorescence, especially for the compounds showing increased FoxP3 levels. Our tool to distinguish highly autofluorescent compounds can help with flow cytometry panel optimization in further validations. With this method, two other compounds identified from the same screen, showing high autofluorescence, were also confirmed to be FoxP3 down-regulators in an optimized flow panel.^32^ In addition, regarding the identification of several autofluorescent compounds with hidden effects as down-regulators on FoxP3, using a different FoxP3 antibody, such as Ax647-FoxP3, in the panel for high-throughput screening can help to avoid compound interference.

The potential candidates identified through flow-cytometry-based screening in T cells may work by either directly or indirectly targeting FoxP3. Compounds that affect FoxP3′s own gene expression, and FoxP3 protein stability, or signaling pathways required for FoxP3 regulation can both result in a change in FoxP3 levels in the flow cytometry assay. Therefore, further efforts are needed to address the mechanisms of action of potential candidates in more detail, understanding their role in FoxP3 regulation or identifying their targets within cells. Indeed, FoxP3 forms complexes with other protein partners to regulate the transcription of genes essential for Treg functions, such as Runx1 and NFAT.^40^ The hits from the phenotypic screen can be functionally investigated *in vitro* by assessing their effects on FoxP3-DNA or FoxP3-protein partner interactions. Both AlphaScreen and surface plasmon resonance (SPR) competitive binding assays can be used to achieve this aim by testing validated hit compounds for their interference with FoxP3-DNA binding and potential FoxP3 protein affinity.^33^ These biochemical assays could be useful for further screenings of small molecules with a functional impact on FoxP3-dependent gene regulation.

From our pilot screen, we identified ethoxyquin as a hit that can down-regulate FoxP3 and inhibit Treg suppressive functions in human primary T cells. Ethoxyquin is an antioxidant compound originally used as a food preservative, and it was later determined to be an Hsp90 inhibitor in other drug screens.^41,42^ Some of the well-known Hsp90 inhibitors, such as ganetespib, were shown to enhance T-cell killing of tumors by reducing the Treg population in the TME.^43^ However, controversial effects of Hsp90 inhibitors were observed, showing an increased percentage of FoxP3^+^ cells and enhanced Treg suppressive functions in mice.^44,45^ We observed a certain direct binding between ethoxyquin or its analogs and Hsp90 protein using SPR analysis (Figure S12A), together with a correlated decrease in protein levels between FoxP3 and Hsp90 in T cells (Figure S12B), which may indicate a mechanism involving Hsp90 to regulate FoxP3 and Treg function. However, the details of the ethoxyquin mode of action should be further elucidated. The identification of active analogs derived from ethoxyquin can help to further understand the mechanism of action based on the compounds’ structure–activity relationship.

In summary, we demonstrate a high-throughput pipeline for screening FoxP3 regulators by the implementation of automated flow cytometry, analysis tools, and validation with cell-based assays. With these methods, we could identify potential candidates with low toxicity and minimal flow cytometry signal interference with optimized antibody panels. Our validation of promising candidates demonstrates the feasibility of screening for FoxP3 regulators in cell-based assays, as well as verifying their effects on Treg functions. The search for analogs based on the hit structure helped us determine the structure–activity relationship. Thus, it enabled us to expand the screen to a larger library and identify additional small molecules with potential for regulating FoxP3^+^ Tregs without introducing toxicity.

## Materials and Methods

### Cell Preparation

Blood samples from human healthy donors were obtained from Oslo University Hospital Blood Bank (Oslo, Norway) with approval from the Regional Ethics Committee (REK #280751) and with donor consent. CD3^+^ T cells were isolated using the RosetteSep T cell enrichment kit (STEM CELL technologies), followed by gradient separation using Lymphoprep (STEM CELL technologies) according to the manufacturer’s instructions. Isolated T cells were frozen in Fetal Bovine Serum (FBS) (Thermo Fisher Scientific) containing 10% DMSO (Sigma-Aldrich) at a concentration of 25 million cells per milliliter in a vial and stored in −80 °C freezers.

Prior to screening, the frozen cells were immediately thawed in a 37 °C water bath until no ice was observed in the suspension. Subsequently, 1 mL of prewarmed FBS was added drop by drop into the vial. The cells were then transferred into a Falcon tube containing 10 mL of prewarmed complete medium and centrifuged at 300 g for 5 min at RT. After washing with medium, the cells were cultured overnight in complete medium at 37 °C in the incubator with 5% CO2. The complete medium used for cell culture consists of RPMI 1640 medium (Thermo Fisher Scientific) supplemented with 10% FBS, nonessential amino acids (NEAA), sodium pyruvate, and 1% penicillin–streptomycin (Thermo Fisher Scientific).

### Cell Distribution and Compound Treatment

For the high-throughput drug screen, the compounds were transferred onto 384-well plates (StorPlate-384 V, PerkinElmer) from stock solutions in DMSO using an acoustic dispenser (Echo 550 or Echo 650, Beckman Coulter), and DMSO in an equivalent volume to the compounds was dispensed into control wells on each plate—minimally eight wells for positive controls, four for negative controls, and four for unstained controls. The Prestwick Chemical Library (PCL, Greepharma) was used in the screen with a final concentration of 10 μΜ for each drug. The library was obtained from the Chemical Biology Platform at the Norwegian Centre for Molecular Biosciences and Medicine (NCMBM) at the University of Oslo.

A total volume of 50 μL of cells per well of cell suspension in complete medium was distributed into preprinted assay plates using a Certus Flex dispenser (Fritz Gyger AG). In brief, cultured cells were collected, resuspended in fresh complete medium, and filtered through a 45 μm strainer (Falcon, Corning) before dispensing. The assay-ready plates were centrifuged at 200 g for 1 min and dispensed with the medium first. Subsequently, 50,000 or 300,000 cells were distributed to the plate to reach a total volume of 50 μL per well. Following this, the plates were centrifuged again at 200 g for 1 min and placed at 37 °C in an incubator with 5% CO_2_ for 24 h.

### Automated Antibody Staining Procedure

All liquid handling procedures for antibody staining were performed using a BioMek i7 pipetting robot (Beckman Coulter) with an integrated plate centrifuge (Agilent). Assay plates were processed as batches of a maximum of four plates per run using 50 μL pipetting tips on the 384-head. All centrifugations were performed at 500 g for 5 min. Removal of supernatants was adjusted to leave a 5 μL residual volume in the wells, with a minimum distance of 0.5 mm m between the tip and the well bottom to prevent disruption of the cell pellets.

After compound treatment, the cells were pelleted by centrifugation, and 45 μL of medium was aspirated from each well. Fixation and permeabilization of cells were then performed using the Human FoxP3 buffer set (BD Biosciences). First, 28 μL of 1x Human FoxP3 Buffer A (1:10 dilution of Buffer A stock in MQ water) was added to the cells for a 10-min incubation at RT (fixation). Subsequently, 10 μL of Human FoxP3 Buffer C (1:14 dilution of Buffer B stock in 1x Buffer A) was added to the cells for another 30-min incubation at RT (permeabilization). The cells were then washed twice with 50 μL of PBS and left within the residual volume of 5 μL of PBS in each well from the last aspiration step.

Antibodies were then transferred with an Echo 550 acoustic dispenser (Beckman Coulter), which dispensed 50 nL of CD4 PE-Cy7 (BD Biosciences) and 75 nL of FoxP3 Horizon V450 (BD Biosciences) into each well. After the antibodies were added, the plates were centrifuged at 500 g for 1 min, followed by shaking for 1 min using a BioTek MultiFlo FX dispenser (Agilent). After 30 min of incubation in the dark at RT, the cells were washed twice with 50 μL of PBS with 2% FBS. After washing, the cells were suspended in a final volume of 20 μL of PBS+2% FBS for flow cytometry.

### High-Throughput Flow Cytometry

High-throughput flow cytometry analysis was conducted on antibody-stained cells in 384-well plates. Cells or beads stained with anti-CD4 PE-Cy7 or anti-FoxP3 Horizon V450 alone, together with nonstained cells, were included for compensation.

The plate, with an initial cell number of 300,000, was analyzed on the BD LSRFortessa (BD Biosciences) in HTS mode, with two sample mixes prior to the recording of 10 μL from each well.

The plates, with an initial cell count of 50,000, were run on an iQue Screener PLUS (Sartorius), which used violet (405 nm) and blue (488 nm) lasers for sample acquisition and measured on all of each laser’s available detectors. The acquisition was performed using the following protocol: initial plate shaking at 2400 rpm for 30 s before the samples were acquired with a sip time of 13.5 s, with the pump speed of 29 rpm. Recording of each well was followed by an additional 1 s “up time” for better well detection. Additional well shakes were performed every 6 wells (4 s at 2400 rpm) and three 1-s rinse cycles with buffer every 12 wells.

### High-Throughput Flow Cytometry Data Process

FACS files obtained from the BD LSRFortessa were analyzed in Cytobank (https://cellmass.cytobank.org) (Beckman Coulter), with file-internal compensation applied.

The file obtained from the iQue screener PLUS is a single document with continuous data from all wells recorded, which requires data segmentation into well-specific individual data. The well-identification process is performed in the iQue ForeCyt software by gating all cells based on the shaking and rinsing intervals described above. Wells stained with the CD4 antibody only, and wells without any antibodies, were used as controls for confirming the well identification. Compensation was then applied to the plates before analysis.

For the gating strategy, lymphocytes and singlets were first gated out, followed by gating on CD4^+^ cells using BL5 on the iQue Screener PLUS (780/60 nm detector) or PE-Cy7 on the BD LSRFortessa, and FoxP3^+^ or FoxP3^–^ cells using VL1 on the iQue Screener PLUS (445/45 nm detector) or Horizon V450 on the BD LSRFortessa, respectively.

### HTS Quality Control and False Positive Filtering

The quality of each plate in the high-throughput flow cytometry assay was determined by calculating the Z′-factor using the formula:

$$Z^{\prime }‐\mathrm{factor}=\mathrm{1 -}\;3(\sigma_{p}\;{+\;\sigma }_{n})/\left|\mu_{p}\;{-\mu }_{n}\right|$$

σ_p_: standard deviation of positive control

σ_n_: standard deviation of negative control

μ_p_: mean of positive controls (FoxP3% in CD4)

μ_n_: mean of negative controls (FoxP3% in CD4)

In each plate, wells containing DMSO were used as controls. Indeed, the wells stained with both FoxP3 and CD4 antibodies were used as positive controls (DMSO), while another four wells stained with the CD4 antibody alone (CD4-only) were included as negative controls.

For filtering toxic compounds, the FSC-H median intensity for each compound well was divided by that of the pooled DMSO control wells on the same plate to obtain a normalized FSC-H MFI (Δratio). Outliers were determined using ±3SD from the mean average Δratio of all compounds in the screen.

For filtering autofluorescent compounds, the V450 MFI of a sample’s FoxP3+ population was divided by the V450 MFI of that sample’s FoxP3- population to obtain a V450 MFI ratio for each sample. Outliers were determined using ±3SD from the mean average V450 MFI ratio of all compounds in the same screen plate.

### Characterization of Compounds in T Cells

Selected candidates were further validated in purified T cells obtained from at least three healthy donors. In brief, 3 × 10^5^ T cells were treated with compounds at specified concentrations, with or without TCR stimulation, using a Human T cell activation/expansion kit (Miltenyi Biotec) at a beads-to-cells ratio of 1:2 for 24–48 h incubation. The harvested cells were washed with PBS and stained with Fixable Viability Dye Alexa Fluoro (Ax)­700 (BD Biosciences), then fixed and permeabilized using Human FoxP3 buffer, followed by flow cytometry staining with anti-CD4 PE-Cy7 and anti-FoxP3 PE or Ax647 (BD Biosciences) antibodies. The selected compounds for validation are given in Table 2.

**Table 2:** The Selected Compounds from the Screen for Validation

Compound id	Compound name	Supplier	Catalog number	Purity
Compound 1	Pralidoxime Chloride	MedChemExpress	HY-B1200	99.83%
Compound 2	Danuorubicin hydrochloride	Sigma (Merck)	D1515	99%
Compound 3	Auranofin	Sigma (Merck)	A6733	98%
Compound 4	Vitamin D3	MedChemExpress	HY-15398	99.94%
Compound 5	Reserpine	MedChemExpress	HY-N0480	99.71%
Compound 6	Benzamil hydrochloride	MedChemExpress	HY-B1546A	99.49%
Compound 7	Ethoxyquin	MedChemExpress	HY-B1425	98.76%

To evaluate the autofluorescent properties of the selected compounds, T cells treated with the specified compounds for 24 h were washed and analyzed using flow cytometry on a BD LSRFortessa with all channels open. Laser channels not interfered with by the compounds were identified and considered for the further design of the subsequent flow cytometry staining panel.

### Treg Functional Assay

For Treg suppression assays, the experiment was performed as previously described.^46^ Tregs and Teffs were purified from healthy donor PBMCs or T cells using the CD4^+^CD25^+^CD127^dim/–^ Regulatory T cell isolation kit (Miltenyi Biotec). Tregs (1× 10^5^) were treated with the indicated compounds under TCR stimulation (beads:cells = 1:2) for 48 h in a 96-V bottom plate. Afterward, the medium containing the compounds was removed after centrifugation, and fresh medium was added before counting the Tregs. Teffs were stained with CellTrace Far Red (Thermo Fischer) and then cocultured with Tregs at a 2:1 ratio (Teff:Treg) in a 96-U bottom plate, under TCR stimulation. After 96 h of incubation, the mixed cells were first stained with Fixable Viability Dye Ax700 and analyzed by flow cytometry to determine Teffs proliferation by calculating the percentage of CellTrace Far Red^+^ cells compared to stimulated Teffs only.

To measure the expression of Treg-specific markers relevant to Treg functions, total T cells were treated with the indicated compounds under TCR stimulation for 48 h. The harvested cells were first washed with PBS, stained with Fixable Viability Dye Ax700, and then fixed and permeabilized by the human FoxP3 buffer, followed by antibody staining for flow cytometry analysis. The antibodies used in the staining panel were FoxP3 PE, ICOS APC-H7/PerCP Cy5.5 (BD Biosciences), LAG-3 BV711, and PD-1 PE-Cy7 (BioLegend). Details of all antibodies used in the flow cytometry analysis are listed in the Supporting Information.

### Analog Search and Validation

Compound analogs of ethoxyquin were identified through similarity searches on Molport (https://www.molport.com/) and ZINC (http://zinc15.docking.org/). The PAINS filter was used to exclude compounds that frequently produce false positives in high-throughput screens. New compounds were selected based on a similarity search with a Tanimoto coefficient cutoff of >0.7. A similarity and distance matrix was generated through a similarity/distance screening process. Multiple rounds of analog searches were performed to identify additional analogs.

The identified analogs of ethoxyquin were ordered through Molport, with detailed information listed in Table 3. Characterization of these compound analogs in T cells and the Treg functional assay was performed as described in the “Characterization of Compounds in T cells” and “Treg Functional Assay” sections above.

**Table 3:** Ethoxyquin Analogs for Validation

Compound	Chemical name	Supplier	Catalog number	Purity
EA1	6-methoxy-2,2,4-trimethyl-1,2-dihydroquinoline	TimTec, LLC	ST002607	>90%
EA2	2,2,4-trimethyl-1,2-dihydroquinolin-6-ol	BLD Pharmatech Ltd.	BD64121	97%
EA3	1-phenyl-2-(2,2,4-trimethyl-1,2-dihydroquinolin-6-yl) oxy) ethanone	TimTec, LLC	ST003336	>90%
EA4	7-tert-butyl-2,2,4-trimethyl-1H-quinolin-6-ol; hydrochloride	ChemBridge Corporation	6097870	>90%
EA6	1-(6-hydroxy-2,2,4-trimethylquinolin-1(2H)-yl) ethan-1-one	ChemDiv, Inc.	Y700–0611	>90%
EA7	2,2,4-trimethyl-1,2-dihydroquinolin-6-yl acetate	BIONET—Key Organics Ltd.	LS-05337	>95%
EA9	6-[4-chlorobenzyl) oxy]-2,2,4-trimethyl-1,2-dihydroquinoline	Vitas M Chemical Limited	STK087182	>90%
EA10	8-methoxy-2,2,4-trimethyl-1,2-dihydroquinoline; hydrochloride	Vitas M Chemical Limited	STK009274	>90%
EA11	2,2,4-trimethyl-1,2-dihydroquinolin-6-yl2,2-dimethylpropanoate	Vitas M Chemical Limited	STK042989	>90%
EA12	6-ethoxy-2,2,4-trimethyl-1,2-dihydroquinoline	Vitas M Chemical Limited	STK772149	>90%
EA13	6-methoxy-1,1,2,2,4-pentamethyl-1,2-dihydroquinolinium	Vitas M Chemical Limited	STL558501	>90%
EA15	2,2,4-trimethyl-1,2-dihydroquinolin-6-yl(4-methoxyphenyl)acetate	Vitas M Chemical Limited	STK508777	>90%
EA16	2,2,4-trimethyl-1,2-dihydroquinolin-6-yl(4-fluorophenyl)acetate	Vitas M Chemical Limited	STK870918	>90%
EA17	2,2,4-trimethyl-1,2-dihydroquinolin-6-ylnaphthalen-1-ylacetate	Vitas M Chemical Limited	STK508570	>90%
EA18	2,2,4-trimethyl-1,2-dihydroquinolin-6-ylbenzoate	Vitas M Chemical Limited	STK040923	>90%
EA19	2,2,4-trimethyl-1,2-dihydroquinolin-6-yl2-methylprop-2-enoate	Vitas M Chemical Limited	STK074617	>90%
EA20	2,2,4-trimethyl-1,2-dihydroquinolin-6-yl2-methylprop-2-enoate	Vitas M Chemical Limited	STK098448	>90%

## Supplementary Material



## Notes

Additional material and methods; supplemental figures including iQue Screener Flow cytometry data analysis (Figure S1); quality controls from the primary screen (Figure S2); autofluorescence check of Compounds **4**–**7** measured by flow cytometry (Figures S3–S6); concentration–response curves for selected candidates (Figure S7); compound structures of validated analogs of ethoxyquin (Figure S8); characterization of ethoxyquin analogs in a cell-based assay (Figure S9); functional validation of ethoxyquin and EA2 (Figure S10); SAR summary of ethoxyquin and its analogs (Figure S11); and effects of ethoxyquin and its analogs on Hsp90 (Figure S12)­(PDF)
