# Supplementary material for: High-Throughput Phenotypic Screen to Identify FoxP3 Regulators in Primary T Cells
Source: ACS Chem Biol. 2026 Apr 3;21(4):764–78. doi: 10.1021/acschembio.5c01019 (PMC13097144; doi:10.1021/acschembio.5c01019)
Supplement: Supplementary file 1 [file cb5c01019_si_001.pdf]

Supporting Information

High-throughput phenotypic screen to identify FoxP3 regulators in primary T cells

Qian Wei[1, 2, *], Ehsan Hajjar[1], Selma Cornillot-Clément[1], Elise Solli[1,3], Nuria García-Díaz[1], Johannes Landskron[4], Alexandra Gade[4], Rafi Ahmad[5], Kjetil Taskén[1, 2, 3, *]

1. Department of Cancer Immunology, Institute for Cancer Research, Oslo University Hospital, 0424 Oslo, Norway

2. Norwegian Centre for Clinical Cancer Research, MATRIX, Division of Cancer Medicine, Oslo University Hospital, 0424 Oslo, Norway

3. Institute of Clinical Medicine, University of Oslo, 0318 Oslo, Norway

4. Norwegian Centre for Molecular Biosciences and Medicine (NCMBM), Nordic EMBL Partnership, University of Oslo, 0318 Oslo, Norway

5. Department of Biotechnology, University of Inland Norway, 2317 Hamar, Norway

* Corresponding authors: Qian Wei, qian.wei@ous-research.no ; Kjetil Taskén, kjetil.tasken@medisin.uio.no

Table of Contents **Page**

**Material and methods**	S2
**Figure S1.** iQue Screener Flow cytometry data analysis.	S3
**Figure S2.** Quality controls in the HTS screen.	S4
**Figure S3.** Autofluorescent check of Compound 4 measured by flow cytometry.	S5
**Figure S4.** Autofluorescent property of Compound 5 measured by flow cytometry	S6
**Figure S5.** Autofluorescent property of Compound 6 measured by flow cytometry.	S7
**Figure S6.** Autofluorescent property of Compound 7 measured by flow cytometry.	S8
**Figure S7.** Concentration-response curves for Compound 4 and Compound 6.	S9
**Figure S8.** List of validated analogs of ethoxyquin.	S10
**Figure S9.** Characterization of ethoxyquin analogs in cell-based assay.	S11
**Figure S10.** Functional validation of ethoxyquin and its analogs.	S12
**Figure S11.** SAR summary of ethoxyquin and its analogs.	S13
**Figure S12.** Effects of ethoxyquin and its analogs on Hsp90.	S14

Material and methods

Functional study in PBMCs

PBMCs were isolated from healthy donors using lymphoprep following manufacture's instructions. Isolated PBMCs were cultured in IMDM (Thermos Fisher Scientific)+5%Human Serum (Sigma) +P/S in 37ºC incubator with 5% CO2.

For the Treg suppression assay using CMV+ donors, the Teff cells were stimulated with PepTivator® CMV pp65 peptide pool (Miltenyi Biotec) prior to co-culture with compound-treated Tregs for 96h.

For immune profiling, PBMCs were treated with compounds for 48h. Harvested cells were stained with viability dye and human Fc receptor binding inhibitor (eBioscience), followed by fixation and permeabilization before further antibody staining.

Flow cytometry antibodies

Anti-human antibodies used in the drug screen and compounds validation are:

CD4 PE-Cy7 (SK3), FoxP3 Horizon V450 (259D/C7), FoxP3 PE (259D/C7), FoxP3 Ax647 (259D/C7), CD8 BV786 (RPA-T8), CD19 PE-Cy7 (H1B13), CD56 BV711(NCAM16.2), HLA-DR APC-H7 (L243), ICOS APC-H7 (DX29) and ICOS PerCP-Cy5.5 (DX29) were ordered from BD Biosciences. CD3 PerCP-Cy5.5 (SK7), CD14 PE (HCD14) and CD16 FITC (B73.1) , LAG-3 BV711 (11C3C65), and PD-1 PE-Cy7 (EH12.2H7) were purchased from BioLegend. Hsp90 Ax647 (F-8) was from Santa Cruz Biotechnology.

Real time (RT)- qPCR

Isolated T cells from healthy donors were stimulated with CD2/3/28 beads and treated with compounds for 48h. Harvested cells were subjected to total RNA isolation using RNeasy Mini Kit (Qiagen). 1µg total RNA was transcribed to cDNA using First Strand cDNA synthesis kit (Thermo Fisher Scientific). RT-qPCR analysis was performed in CFX Duet Real-Time PCR system (Bio-Rad) using SYBR green master mix (Thermo Fisher Scientific). Quantitative gene expression analysis was calculated using the $2^{-\Delta CCT}$ method. RPS9 was used as internal control, and the fold-change of FoxP3 gene expression in compound-treated samples was obtained by normalization to DMSO control. Primers used for qPCR are: FoxP3- Forward 5'-GAACGCCATCCGCCACAACCTG-3', Reverse 5'- CCCTGCCCCCACCACCTCTGC-3'; RPS9- Forward 5'-CGAAGGGTCTCCGCGGGGTCACAT-3', Reverse 5'-CGAAGGGTCTCCGCGGGGTCACAT-3'.

Surface plasmon resonance (SPR) analysis

Purified human His-Hsp90 protein (MedChem Express) was immobilized on a CM5 chip (Cytiva) using amine coupling method in Biacore T200 (Cytiva). Indeed, 50 µg/ml His-Hsp90 was injected to be immobilized on flow cell 2, with flow cell 1 as blank control, using 1x HBS-EP+ as running buffer (Cytiva).

To measure the affinity of compounds to Hsp90, compounds at 50µM were ran as analytes according to the LMW (low molecular weight) screening programme in Biacore T200, using PBS+P+(0.05% Tween 20)+2%DMSO as running buffer. Serial dilutions of DMSO at 1.5%–3% range were used for solvent correction. The binding between compound and Hsp90 is measured as response units (RU) after reference subtraction from control flow cell and solvent correction. In the SPR LMW programme, binding (RU) is collected at 48s (binding_early) during injection and 108s (binding_late) after injection, representing association and dissociation phases of the small molecule binding to Hsp90.

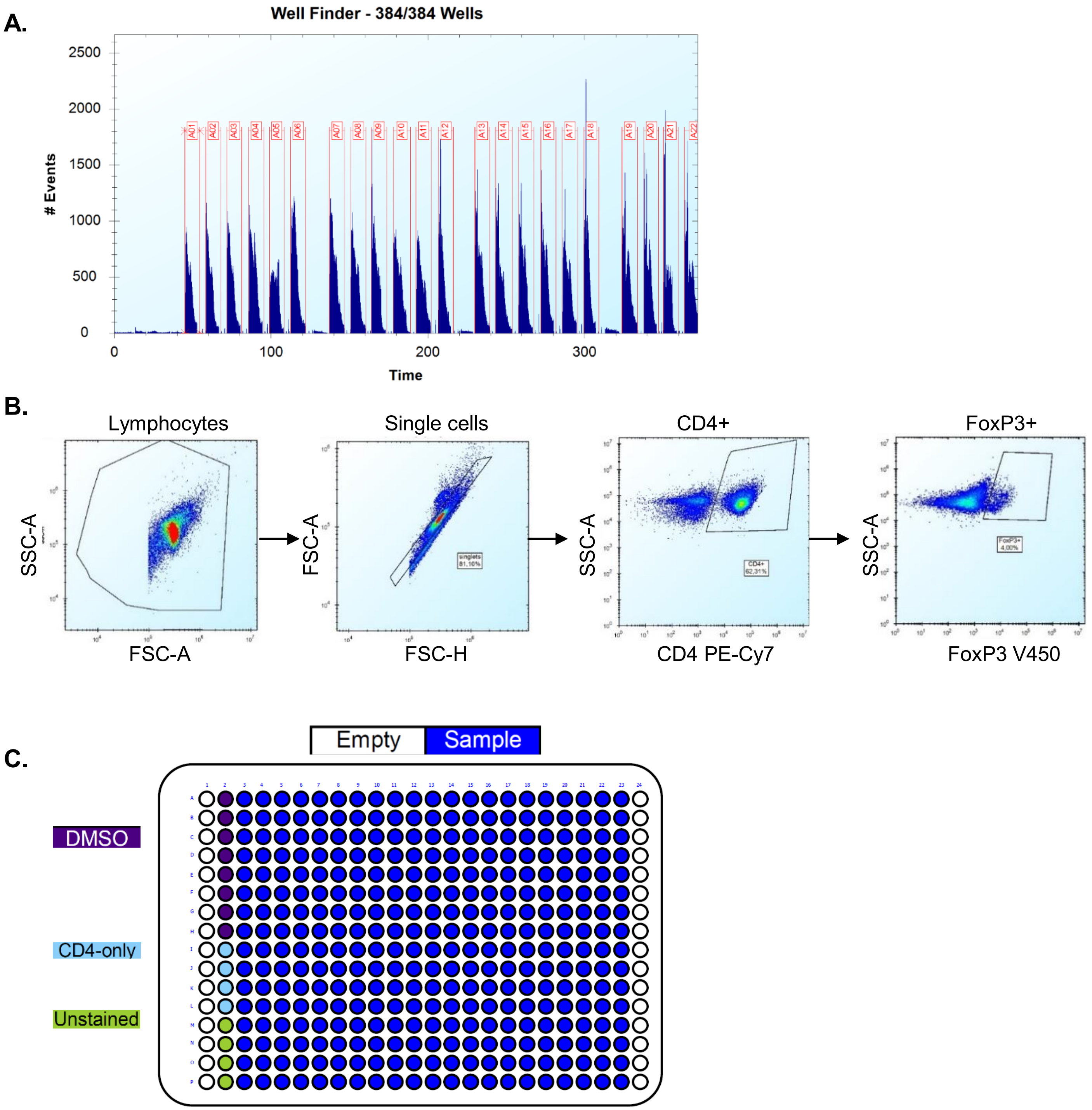

Figure S1. iQue Screener Flow cytometry data analysis.

High-throughput flow cytometry data collected from the iQue screener PLUS were processed using iQue Forecyt® software (Sartorius). **A)** Well-identification was performed within the software. The well-ID was determined by the record time, allowing for re-verification through the wash interval settings. **B)** The gating strategy in Forecyt is shown. **C)** The layout of a 384-well plate design for iQue screener is represented, highlighting different groups in distinct colors.

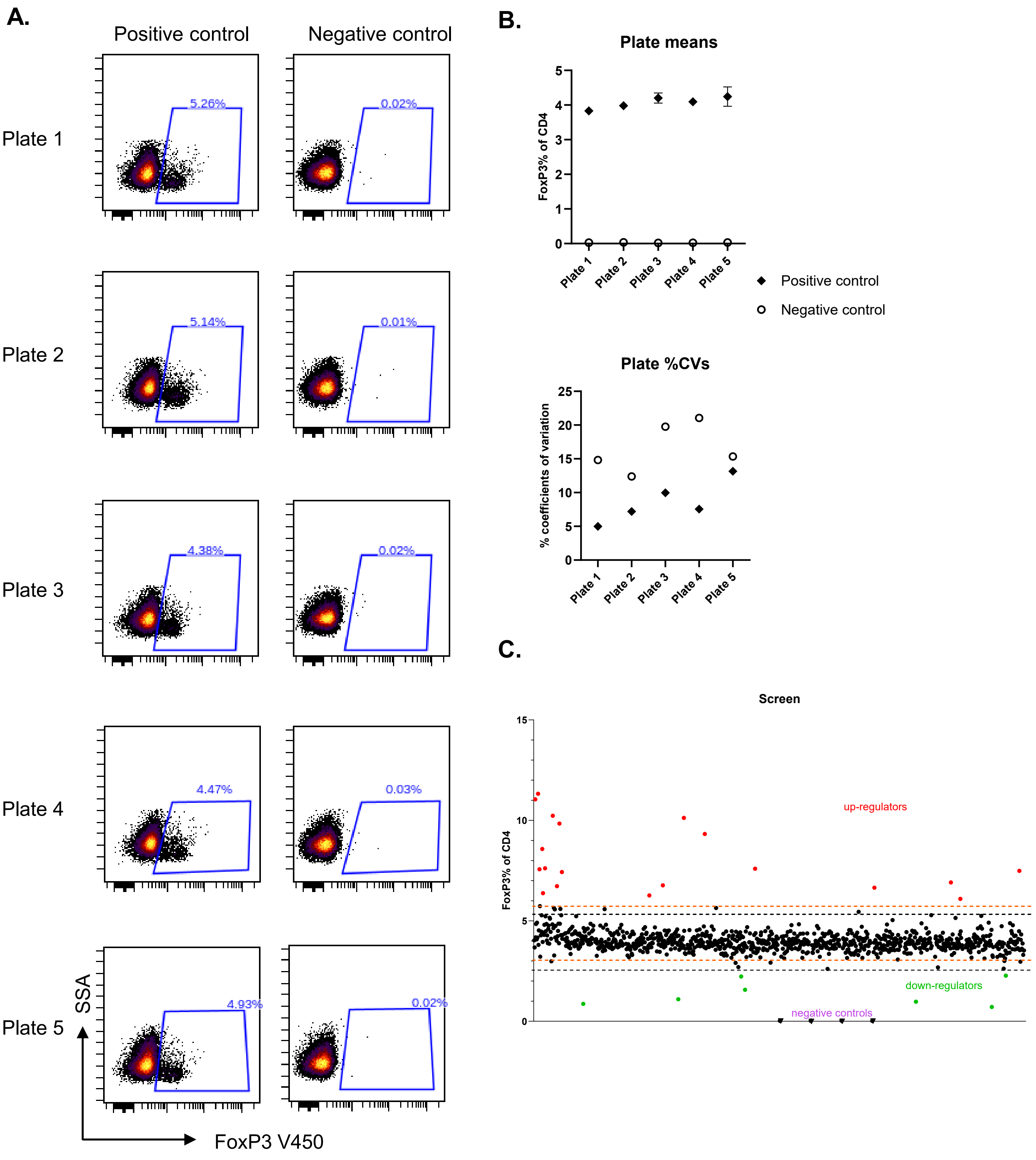

Figure S2. Quality controls in the HTS screen.

A) The scattered plots from cytometry analysis show the representative positive controls and negative controls used for Z'-facotr calculation from each plate in the screening of Prestwick library. The gating indicates the percentage of FoxP3 in CD4$^+$ T cells. **B)**. The graph represents the summary of both positive and negative controls in each screening plate, by calculating the mean of FoxP3% in CD4$^+$ T cells and % coefficients of variation (CV). **C)**. The graph shows the original FoxP3% data in all screening plates, with the lines marking the threshold for hit selection related to Figure 2C.

A.

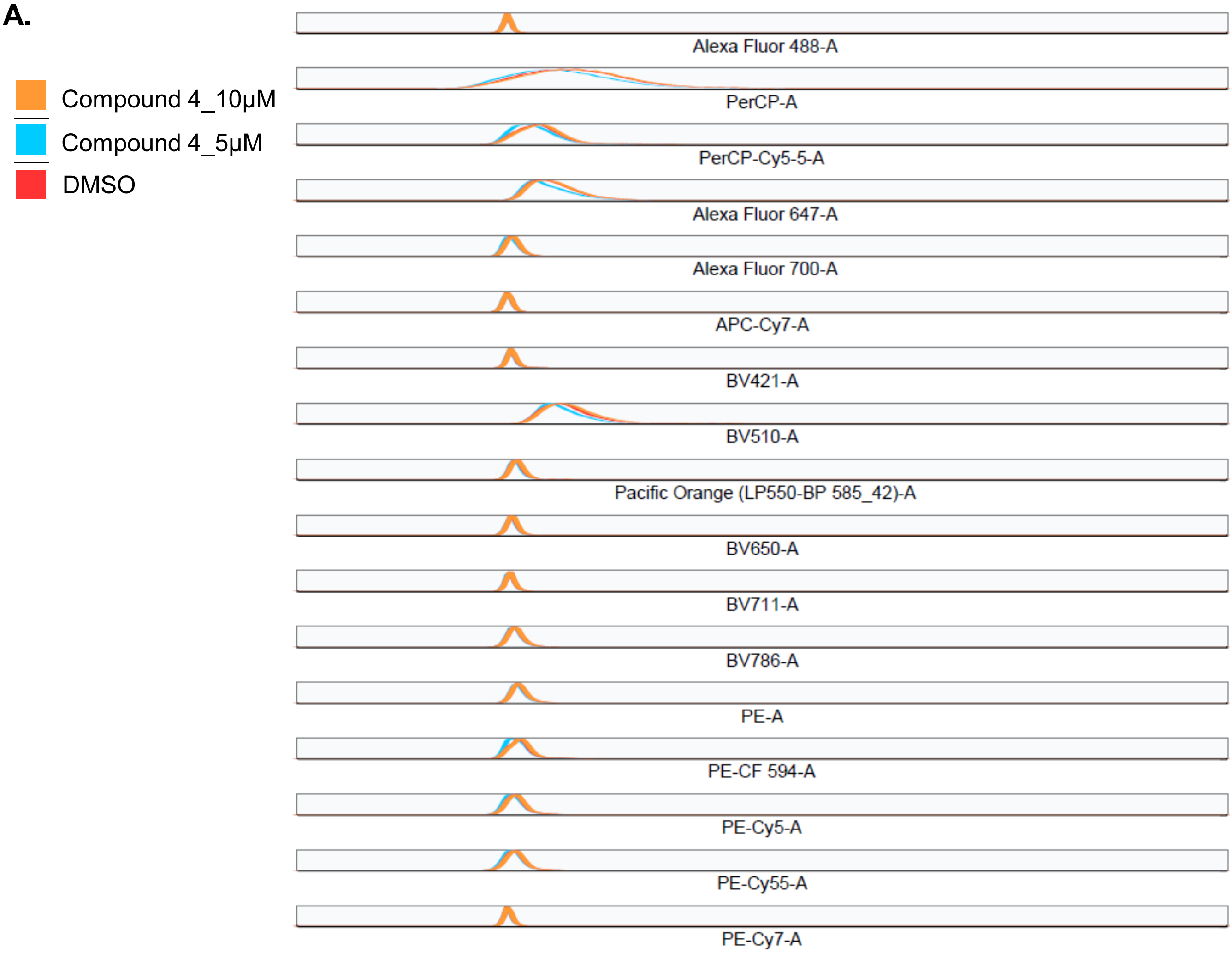

B.

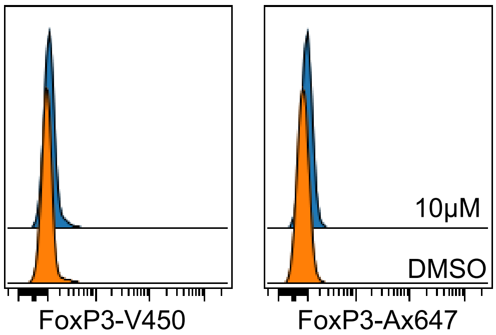

Figure S3. Autofluorescent check of Compound 4 measured by flow cytometry.

A) T cells were treated with DMSO, or with Compound 4 at concentrations of 5 or 10 μM for 24 h, followed by flow cytometry analysis using BD LSRFortessa with all channels open. The histograms illustrate the overlay of each channel between the DMSO control and compound-treated samples. **B)** T cells were treated with Compound 4 at 10 μM for 24 h, followed by fixation and permeabilization before antibody staining. Flow cytometry analysis was then performed by staining the cells with CD4 and FoxP3 antibodies. Histograms represent the staining patterns of FoxP3-V450 or FoxP3-Ax647 in CD4+ T cells in compound treated cells compared to DMSO control.

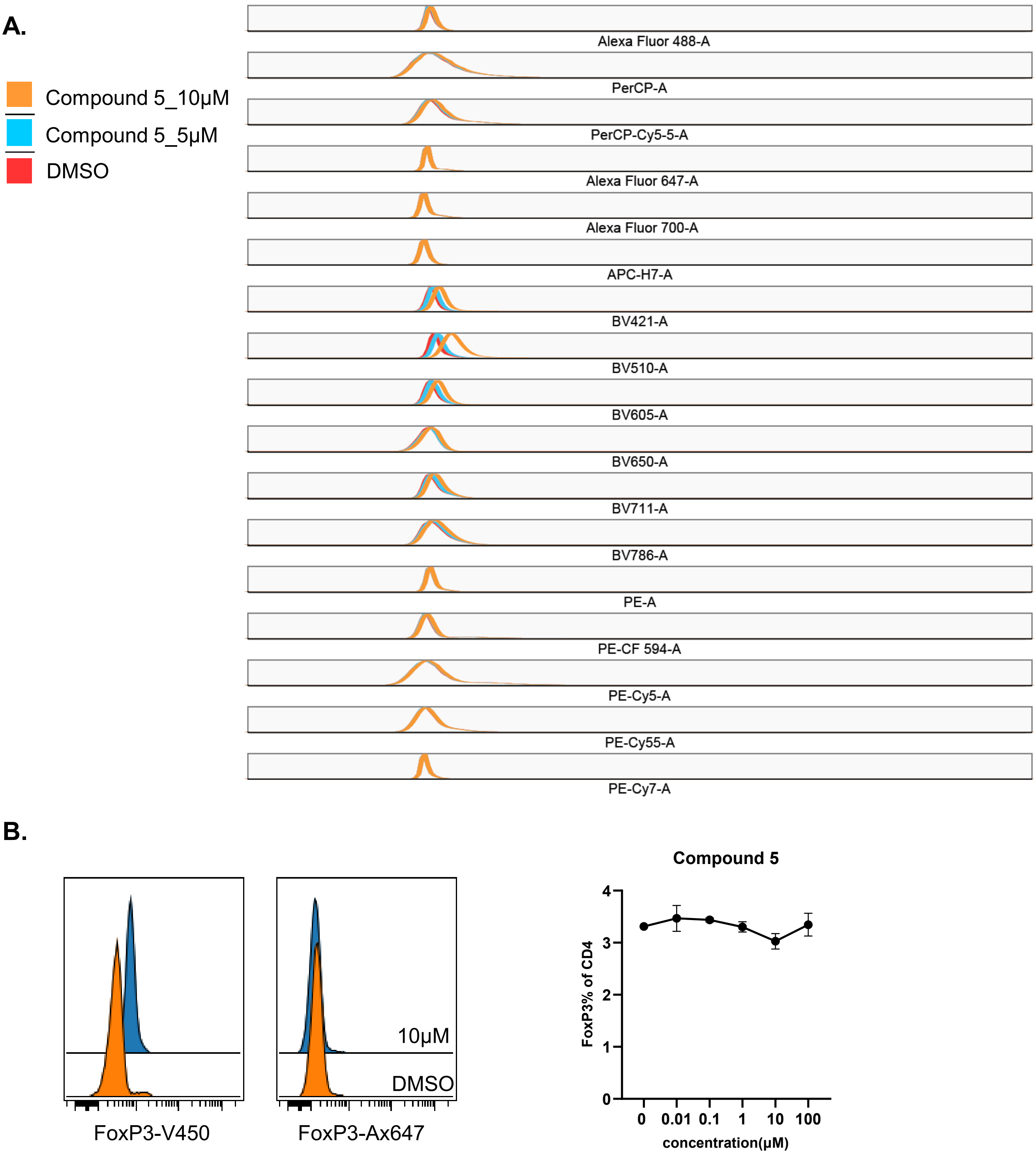

Figure S4. Autofluorescent property of Compound 5 measured by flow cytometry.

A) T cells were treated with DMSO, or with Compound 5 at concentrations of 5 or 10 μM for 24 h, followed by flow cytometry analysis using BD LSRFortessa with all channels open. The histograms illustrate the overlay of each channel between the DMSO control and compound-treated samples. **B)** T cells were treated with Compound 5 at 10 μM for 24 h, followed by fixation and permeabilization before antibody staining. Flow cytometry analysis was then performed by staining the cells with CD4 and FoxP3 antibodies. Histograms represent the staining patterns of FoxP3-V450 or FoxP3-Ax647 in CD4+ T cells in compound treated cells compared to DMSO control. The graph presents the percentage of FoxP3-Ax647 in CD4^{+} T cells treated with Compound 5 at different concentrations. Data are represented as mean ± SEM (n=3 healthy donors).

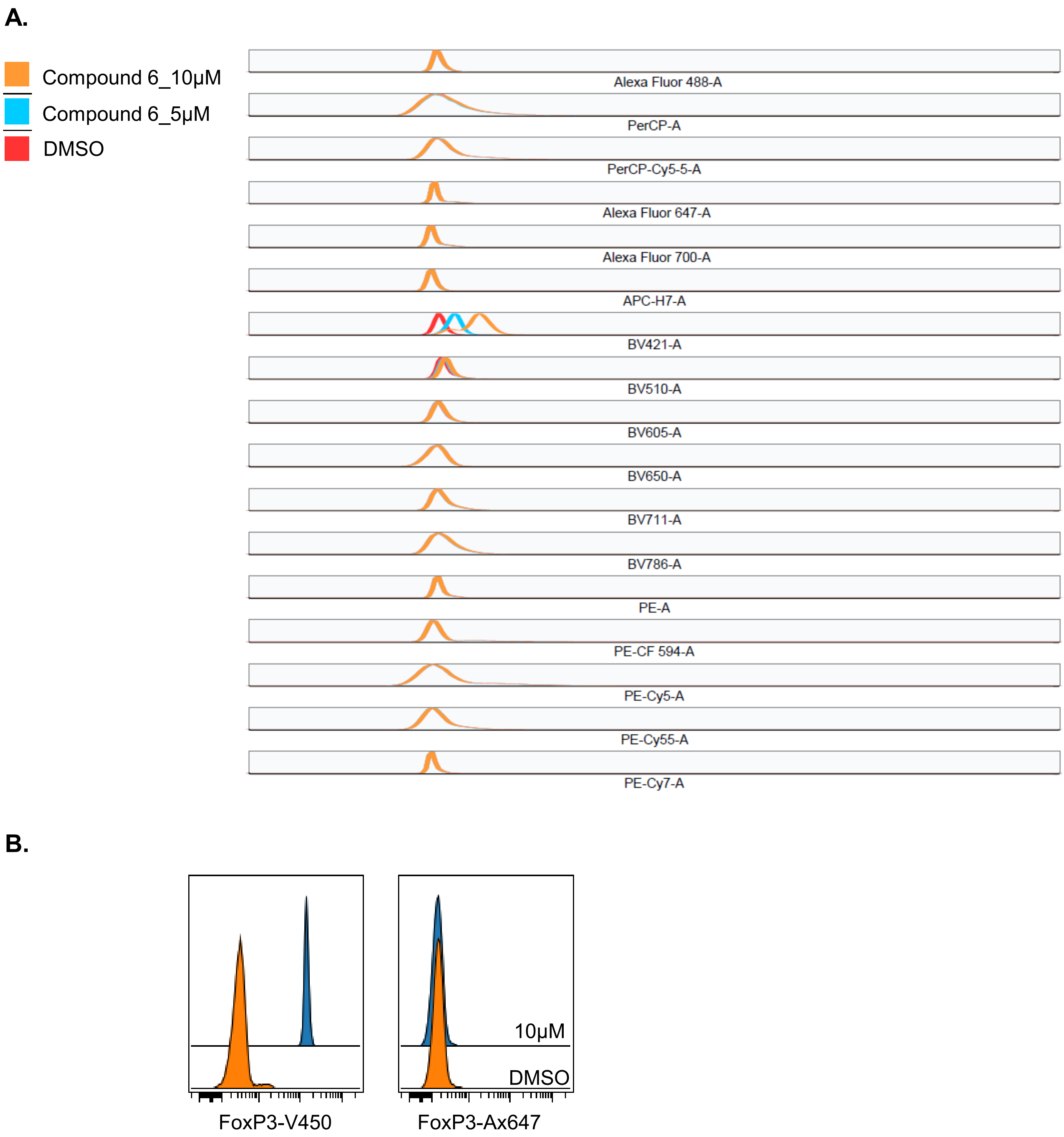

Figure S5. Autofluorescent property of Compound 6 measured by flow cytometry.

A) T cells were treated with DMSO, or Compound 6 at 5 or 10 μM for 24 h and then subjected to flow cytometry analysis on BD LSRFortessa with all channels open. Histograms illustrate the overlay of each channel between DMSO and the compound-treated samples.

B) T cells were treated with Compound 6 at 10 μM for 24 h, followed by flow cytometry analysis using anti-CD4 and anti-FoxP3 antibodies. Histograms represent the staining patterns of FoxP3-V450 or FoxP3-Ax647 in CD4+ T cells in compound treated cells compared to DMSO control.

A.

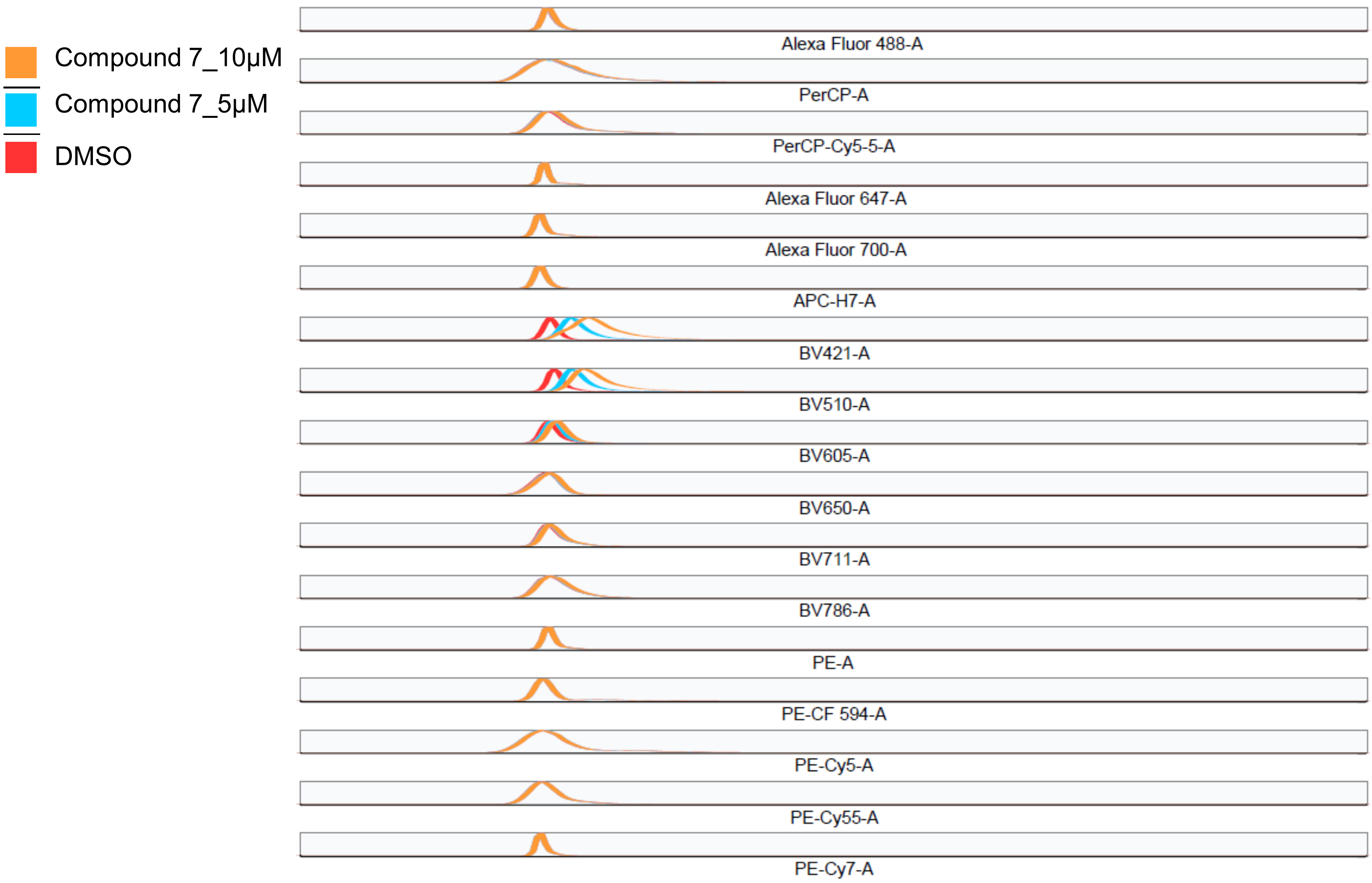

B.

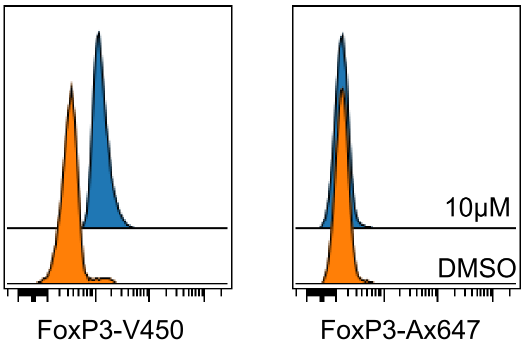

Figure S6. Autofluorescent property of Compound 7 measured by flow cytometry.

A) T cells were treated with DMSO, or Compound 7 at 5 or 10 μM for 24 h and then subjected to flow cytometry analysis on BD LSRFortessa with all channels open. Histograms illustrate the overlay of each channel between DMSO and the compound-treated samples.

B) T cells were treated with Compound 7 for 24h before flow cytometry analysis by staining with anti-CD4 and anti-FoxP3 antibodies. Histograms represent the staining patterns of FoxP3-V450 or FoxP3-Ax647 in CD4+ T cells in compound treated cells compared to DMSO control.

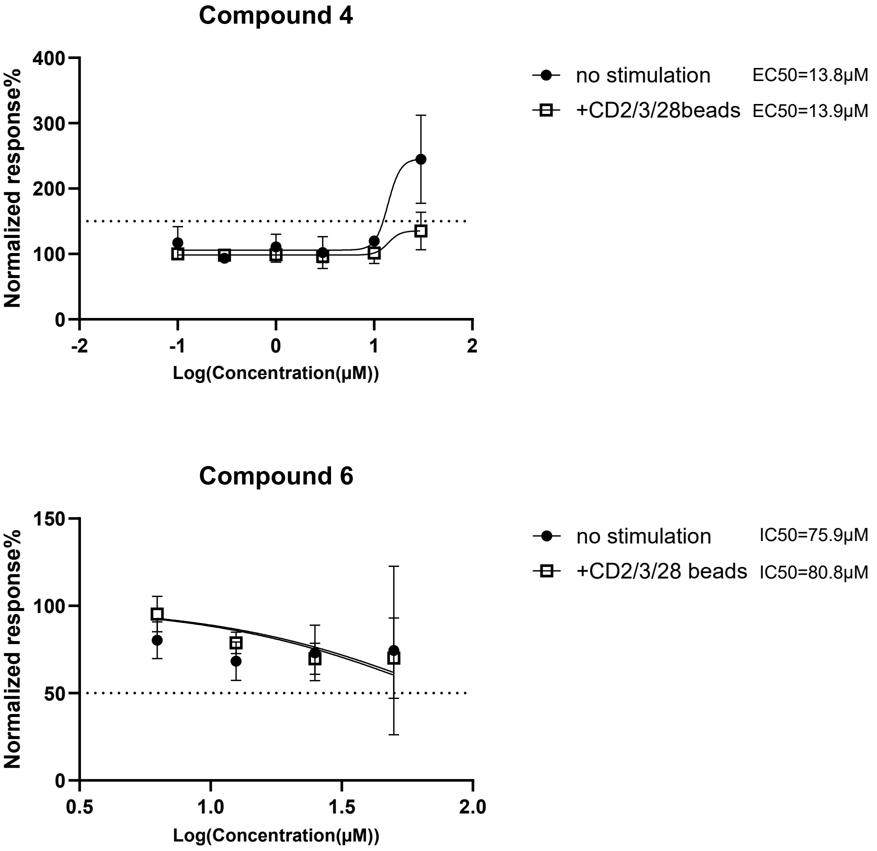

Figure S7. Concentration-response curves for Compound 4 and Compound 6.

CD3$^+$ T cells were treated with compounds with or without TCR stimulation at the indicated concentrations for 24 h, followed by flow cytometry analysis. Normalized response: the percentage of FoxP3 in CD4$^+$ T cells in the compound-treated samples was normalized to the DMSO control which was set to 100%. EC50 or IC50 was calculated by fitting dose-dependent curves in Graphpad. (n=3 donors)

A.

EA4

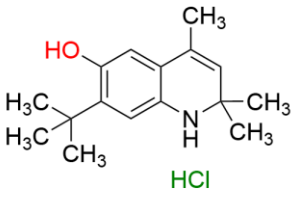

EA6

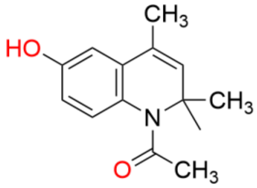

EA7

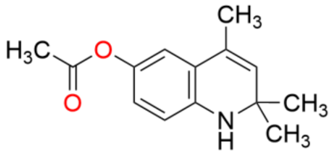

EA9

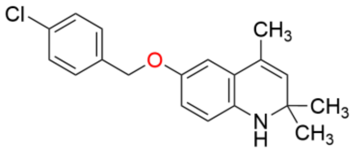

EA10

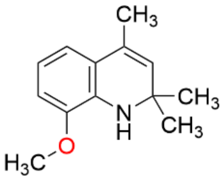

EA11

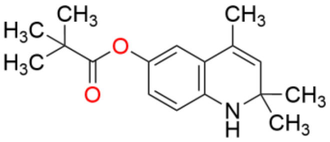

EA12

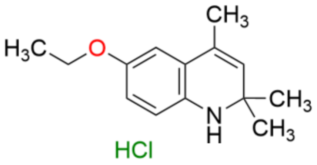

EA13

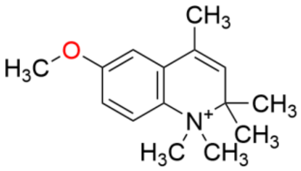

B.

EA15

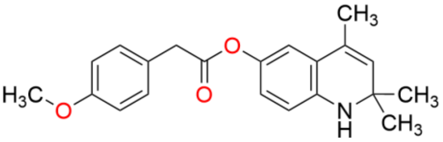

EA16

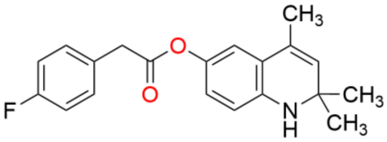

EA17

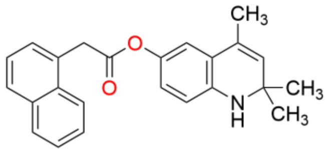

EA18

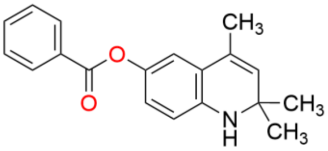

EA19

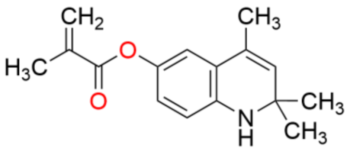

EA20

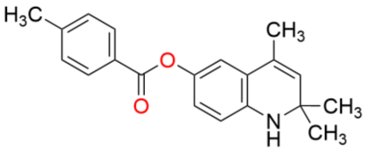

Figure S8. List of validated analogs of ethoxyquin.

Compound structures of ethoxyquin analogs derived from EA2 **(A)** and EA3 **(B)** .

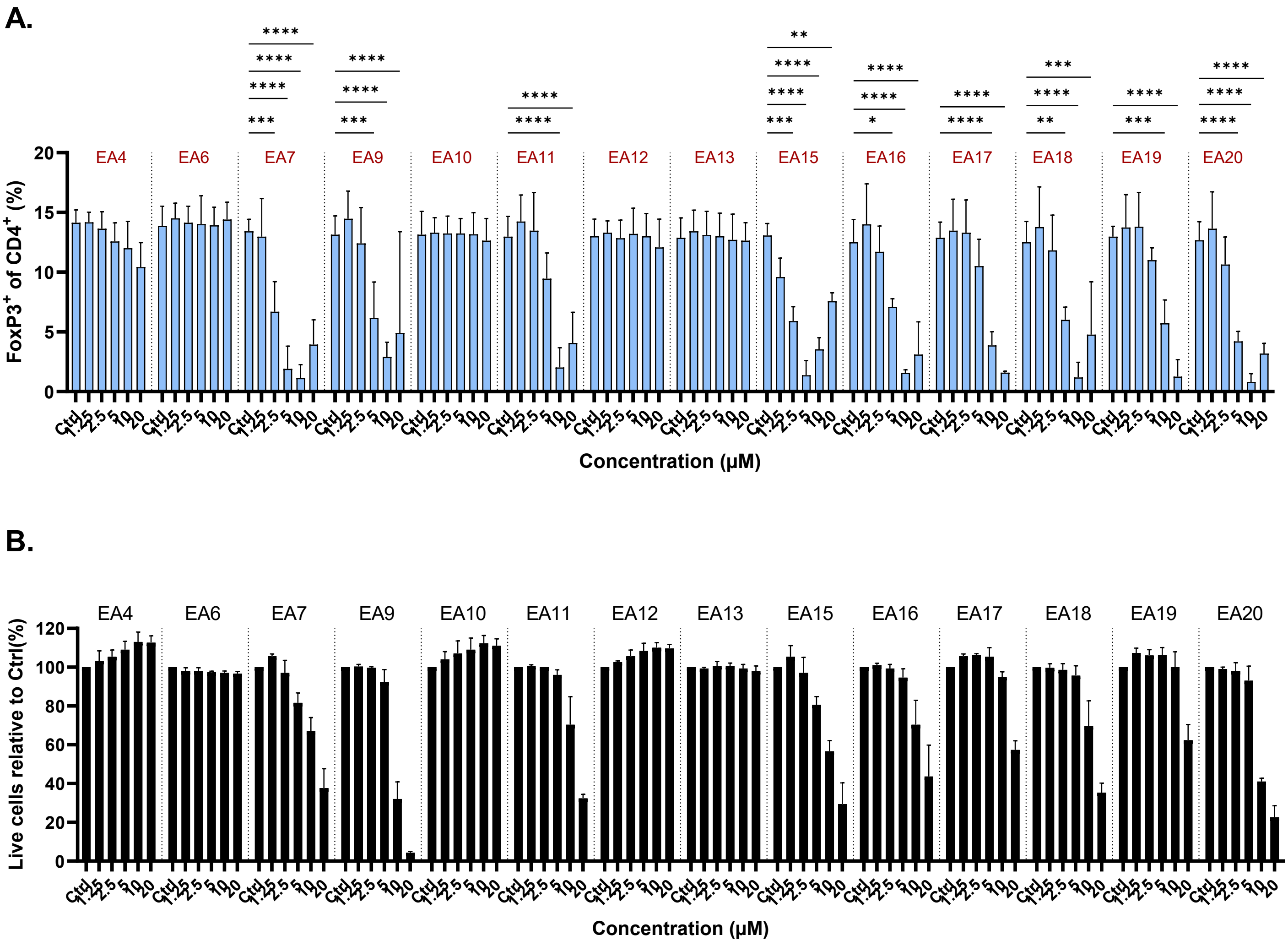

Figure S9. Characterization of ethoxyquin analogs in cell-based assay.

$CD3^+$ T cells were treated with specified compounds under TCR stimulation at the indicated concentrations for 48h, followed by viability dye and antibody staining for flow cytometry analysis. **A)** The graph shows the percentage of FoxP3 in $CD4^+$ T cells from samples under different treatments. **B)** The percentage of live cells in compound-treated cells at the indicated concentrations were normalized to the DMSO control which is set to 100%. Normalized percentage of live cells relative to DMSO control is shown. Data are represented as mean ± SEM (n=3 healthy donors). * p<0.5, ** p <0.05, *** p <0.005, **** p <0.0005, 2-way ANOVA.

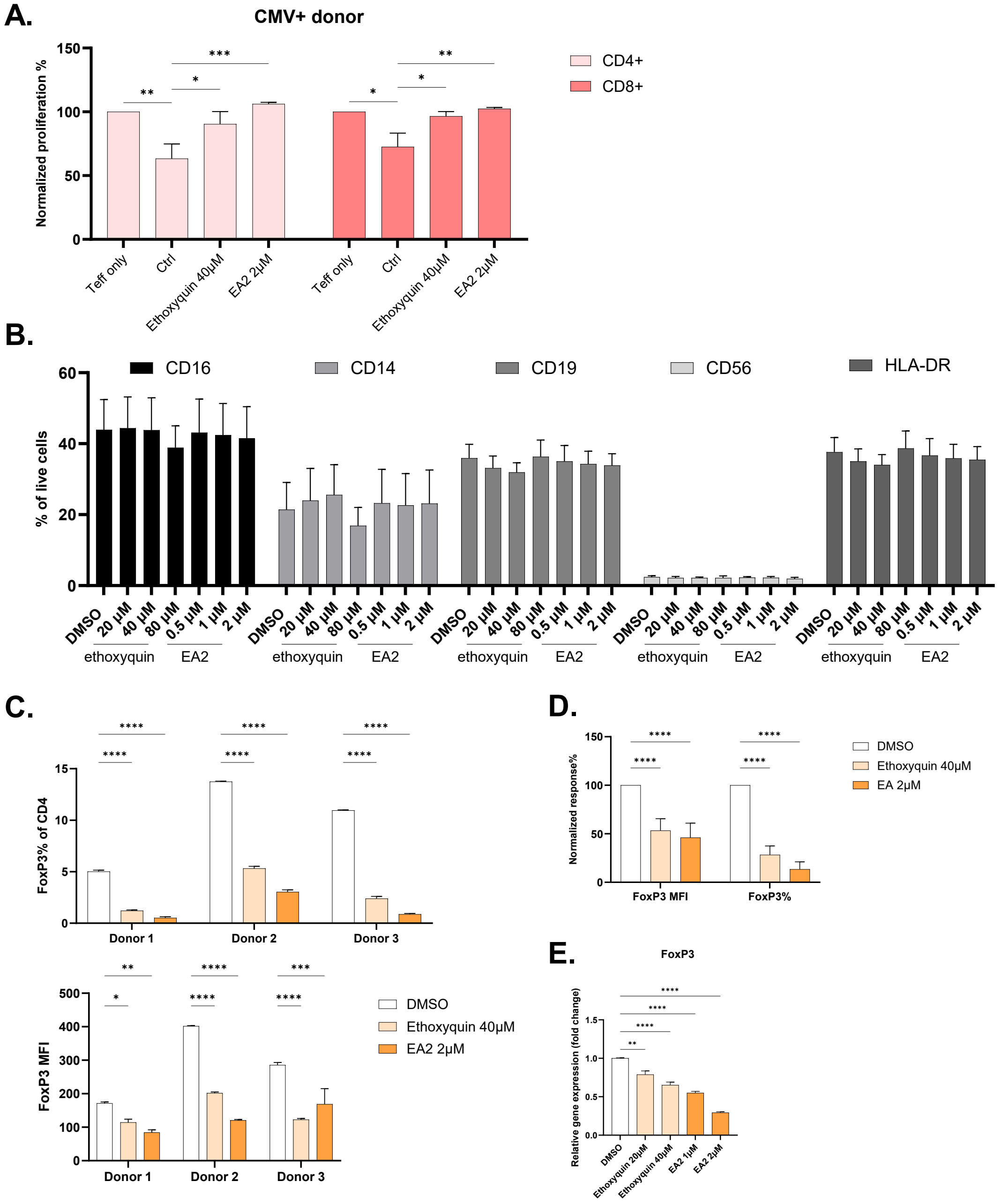

Figure S10. Functional validation of ethoxyquin and EA2.

A) Treg suppression assay in CMV+ donors. Isolated Tregs were treated with indicated compounds for 48h, then co-cultured for 96h with CellTrace Far Red-labeled, CMV peptide activated Teff before harvesting for flow cytometry analysis. The proliferation of $CD4^+$ and $CD8^+$ Teffs was determined by measuring the percentage of CellTrace Far Red^+ cells among live cells, which was normalized to the Teff only. Data are represented as mean ± SEM (n=3 donors). **B)** PBMCs from healthy donors were treated with compounds at indicated concentrations for 48h, followed by flow cytometry analysis to determine their effects on non-T cell populations. Data are represented as mean ± SEM (n=3 healthy donors). **C-E)** TCR-stimulated $CD3^+$ T cells were treated with compounds for 48h, followed by flow cytometry **(C,D)** or RT-qPCR **(E)** analysis to measure FoxP3 expression. **C)** Graphs show the percentage of FoxP3 in CD4 T cells (upper) or FoxP3 median intensity (MFI) (lower) from each individual donor (error bars show SEM between technical replicates, n=3). **D**) The graph shows the pooled data from 3 donors, representing the normalized response in the compound-treated groups compared to DMSO, which was set as 100%. Data are represented as mean±SEM and analyzed using a mixed model, n=3 donors. **E)** The graph represents relative FoxP3 gene expression normalized to RPS9 internal control in the compound-treated groups compared to DMSO control. Data are represented as mean ± SEM (n=3 replicates). * p<0.5, ** p <0.05, *** p <0.005, **** p <0.0005, 2-way ANOVA (**A**,**C**,**D**) or one-way ANOVA (**E**).

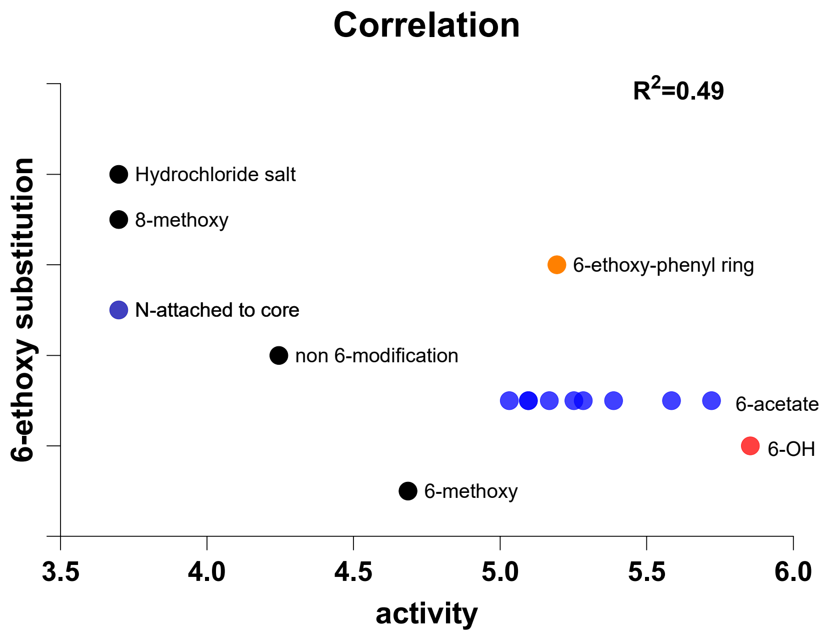

Figure S11. SAR summary of ethoxyquin and its analogs.

Correlation analysis was performed in GraphPad between activity and 6-ethoxy substitution of all ethoxyquin analogs listed in Table 1. Activity of each compound is converted from its IC50 (μM) using the formula: activity = -log10 ($IC50 \times 10^{-6}$). Each blue dot represents a single compound with the core 6-acetate modification (from left to right: EA19, EA17, EA3, EA11, EA16, EA18, EA20, EA7, EA15). The red dot represents the compound with 6-OH substitution (EA2). The orange dot represents the compound adding a phenyl ring to the core 6-ethoxy group (EA9). The rest dots represent compounds with indicated modification.

A.

Compound	Response Unit (binding_early)	Response Unit (binding_late)
Ethoxyquin	2.5	1.8
EA1	1.8	1.6
EA2	2.4	2
EA3	11	5.2
EA4	6.3	4.7
EA6	2.3	2
EA7	2	1.8
EA9	0.6	-7.8
EA10	2.5	2.1
EA11	4.1	3
EA12	2.7	3
EA13	2.2	2.2
EA15	29.1	15.6
EA16	15.6	8.3
EA17	36.5	23.8
EA18	29.9	15.1
EA19	3.1	2.6
EA20	7.4	4.2

B.

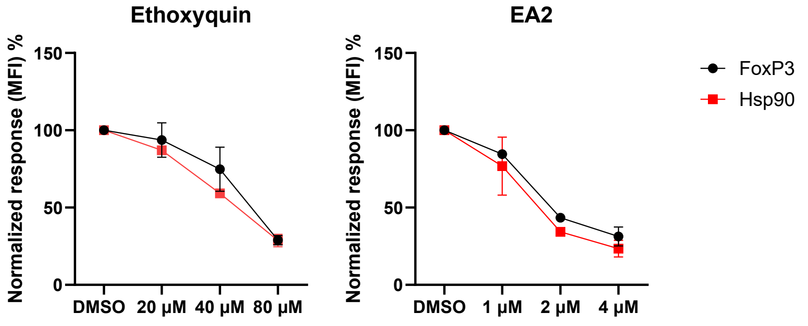

Figure S12. Effects of ethoxyquin and its analogs on Hsp90. A) SPR LMW screen assay was performed to determine the binding of compounds listed to Hsp90. Response units (RU) represent level of binding of compound to Hsp90 protein that is immobilized on a CM5 chip in the association and dissociation phases (binding_early, binding_late). **B)** T cells were treated with indicated compounds for 48h under TCR stimulation. Flow cytometry analysis was performed to measure FoxP3 and Hsp90 expression (MFI). Data represents the normalized response of MFI in compound-treated samples compared to DMSO control which is set as 100% (n=3 healthy donors).
